# Genome-wide analysis of self-reported risk-taking behaviour and cross-disorder genetic correlations in the UK Biobank cohort

**DOI:** 10.1038/s41398-017-0079-1

**Published:** 2018-02-02

**Authors:** Rona J. Strawbridge, Joey Ward, Breda Cullen, Elizabeth M. Tunbridge, Sarah Hartz, Laura Bierut, Amy Horton, Mark E. S. Bailey, Nicholas Graham, Amy Ferguson, Donald M. Lyall, Daniel Mackay, Laura M. Pidgeon, Jonathan Cavanagh, Jill P. Pell, Michael O’Donovan, Valentina Escott-Price, Paul J. Harrison, Daniel J. Smith

**Affiliations:** 1 Institute of Health and Wellbeing, University of Glasgow, Glasgow, UK; 2 Department of Medicine Solna, Karolinska Institutet, Stockholm, Sweden; 3 Department of Psychiatry, University of Oxford, Oxford, UK; 4 Oxford Health NHS Foundation Trust, Oxford, UK; 5 Department of Psychiatry, Washington University School of Medicine in St Louis, St Louis, MO USA; 6 Transmontane Analytics, Tuscon, AZ USA; 7 School of Life Sciences, College of Medical, Veterinary and Life Sciences, University of Glasgow, Glasgow, UK; 8 MRC Centre for Neuropsychiatric Genetics and Genomics, Cardiff University, Cardiff, UK

## Abstract

Risk-taking behaviour is a key component of several psychiatric disorders and could influence lifestyle choices such as smoking, alcohol use, and diet. As a phenotype, risk-taking behaviour therefore fits within a Research Domain Criteria (RDoC) approach, whereby identifying genetic determinants of this trait has the potential to improve our understanding across different psychiatric disorders. Here we report a genome-wide association study in 116,255 UK Biobank participants who responded yes/no to the question “Would you consider yourself a risk taker?” Risk takers (compared with controls) were more likely to be men, smokers, and have a history of psychiatric disorder. Genetic loci associated with risk-taking behaviour were identified on chromosomes 3 (rs13084531) and 6 (rs9379971). The effects of both lead SNPs were comparable between men and women. The chromosome 3 locus highlights *CADM2*, previously implicated in cognitive and executive functions, but the chromosome 6 locus is challenging to interpret due to the complexity of the HLA region. Risk-taking behaviour shared significant genetic risk with schizophrenia, bipolar disorder, attention-deficit hyperactivity disorder, and post-traumatic stress disorder, as well as with smoking and total obesity. Despite being based on only a single question, this study furthers our understanding of the biology of risk-taking behaviour, a trait that has a major impact on a range of common physical and mental health disorders.

## Introduction

Risk-taking behaviour is an important aspect of several psychiatric disorders, including attention-deficit hyperactivity disorder (ADHD)^1,2^ and bipolar disorder (BD)^3^, as well as problem behaviours such as smoking and drug and alcohol misuse^4,5^. The link between risk-taking behaviour and schizophrenia (SCZ) is more complex, with difficulties in conditional reasoning^6^, problems with delayed gratification and poor impulse control occurring alongside more conservative risk assessment^7^. Physical health problems such as obesity might also be considered to be related to increased propensity towards risk taking: obesity includes aspects of aberrant reward processing, response inhibition, and decision making^8^. The Research Domain Criteria (RDoC) approach suggests that studying dimensional psychopathological traits (rather than discrete diagnostic categories), as well as relevant traits across the whole spectrum (“normal” through to pathological) of the population may be a more useful strategy for identifying biology, which cuts across psychiatric diagnoses^9^. In this respect, risk-taking behaviour is an important phenotype for investigation. It may also be useful for investigating the overlap between psychiatric disorders and conditions such as obesity and smoking.

To date, an association between a locus on chromosome 3 and risk-taking behaviour has been published^10,11^, but no genome-wide genetic study with a primary focus on risk-taking behaviour has been conducted. Genome-wide association studies (GWAS) of related phenotypes, such as impulsivity and behavioural disinhibition, have so far been underpowered for detecting associations at a genome-wide level. Here we conduct a primary GWAS of self-reported risk-taking behaviour in 116,255 participants from the UK Biobank cohort. We use expression quantitative trait loci analysis to highlight plausible candidate genes and we assess the extent to which there is a genetic correlation between risk-taking and several mental and physical health disorders, including ADHD, SCZ, BD, major depressive disorder (MDD), anxiety, post-traumatic stress disorder (PTSD), smoking status (ever smoker), lifetime cannabis use, fluid intelligence, years of education, obesity, and alcohol use disorder.

## Materials and methods

### Sample

UK Biobank is a large population cohort, which aims to investigate a diverse range of factors influencing risk of diseases, which are common in middle and older age. Between 2006 and 2010, >502,000 participants (age range from 40 and 69 years) were recruited from 22 centres across the United Kingdom (UK)^12^. Comprehensive baseline assessments included social circumstances, cognitive abilities, lifestyle, and measures of physical health status. The present study used the first release of genetic data on approximately one-third of the UK Biobank cohort. In order to maximise homogeneity, we included only participants of (self-reported) white UK ancestry.

Informed consent was obtained by UK Biobank from all participants. This study was carried out under the generic approval from the NHS National Research Ethics Service (approval letter dated 13 May 2016, ref 16/NW/0274) and under UK Biobank approval for application #6553 “Genome-wide association studies of mental health” (PI Daniel Smith).

### Genotyping, imputation, and quality control

The first release of genotypic data from UK Biobank, in June 2015, included 152,729 UK Biobank participants. Samples were genotyped with either the Affymetrix UK Biobank Axiom array (Santa Clara, CA, USA; approximately 67%) or the Affymetrix UK BiLEVE Axiom array (33%), which share at least 95% of content. Autosomal data only were available.

Imputation of the data has previously been described in the UK Biobank interim release documentation^13^. In brief, single-nucleotide polymorphisms (SNPs) were excluded prior to imputation if they were multiallelic or had minor allele frequency (MAF) <1%. A modified version of SHAPEIT2 was used for phasing and IMPUTE2 (implemented on a C++ platform) was used for the imputation^14,15^. A merged reference panel of 87,696,888 biallelic variants on 12,570 haplotypes constituted from the 1000 Genomes Phase 3 and UK10K haplotype panels^16^ was used as the basis for the imputation. Imputed variants with MAF < 0.001% were filtered out of the data set used for subsequent analysis.

The Wellcome Trust Centre for Human Genetics applied stringent quality control, as described in UK Biobank documentation^17^, before release of the genotypic data set. UK Biobank genomic analysis exclusions were applied (Biobank Data Dictionary item #22010). Participants were excluded from analyses due to relatedness (#22012: genetic relatedness factor; one member of each set of individuals with KING-estimated kinship coefficient >0.0442 was removed at random), sex mismatch (reported compared with genetic) (#22001: genetic sex), non-Caucasian ancestry (#22006: ethnic grouping; self-reported and based on principal component (PC) analysis of genetic data), and quality control failure (#22050: UK BiLEVE Affymetrix quality control for samples and #22051: UK BiLEVE genotype quality control for samples). SNPs were removed due to deviation from Hardy–Weinberg equilibrium at *p* < 1 × 10^−6^, MAF < 0.01, imputation quality score <0.4 and >10% missingness in the sample after excluding genotype calls made with <90% posterior probability.

The second release of genetic data from the UK Biobank (July 2017) included a further 349,935 samples. Genotyping platforms, quality control, and pre-imputation procedures were consistent with the first data release. Imputation of genotypes at additional SNP loci for all participants (*n* = 502,664) was carried out using the Haplotype Reference Consortium (HRC) reference panel, and post-imputation quality control was consistent with that of the first data release.

### Risk-taking phenotype

The baseline assessment (2006–2010) of UK Biobank participants included the question “Would you describe yourself as someone who takes risks?” (data field #2040), to which participants replied yes or no. Individuals who responded “yes” to the risk-taking question are here referred to as “risk takers” and those who responded “no” are here referred to as “not risk takers or controls”. For a subset of participants, the same question (“Would you describe yourself as someone who takes risks?”) was asked at follow-up (2012–2013), enabling an assessment of response consistency.

### Discovery analyses

A total of 116,255 individuals and 8,781,003 variants (first data release) were included in the discovery analysis. A total of 29,703 participants were classed as risk takers and 86,552 were controls. Association analysis was conducted in PLINK^18^ using logistic regression, assuming a model of additive allelic effects and models were adjusted for sex, age, genotyping array, and the first eight genetic PCs (Biobank Data Dictionary items #22009.01 to #22009.08) to control for hidden population stratification. The threshold for GWAS significance was set at *p* < 5 × 10^–8^. Demographics of the discovery sample set are presented in Table 1. For quality control purposes, a GWAS of the individuals included in the discovery analysis was run with the second release genetic data (HRC-imputed) and using the updated genetic exclusions and covariates used. Using the updated exclusions resulted in a slight increase in the number of individuals included in the analysis: *n* = 117,755, of whom *n* = 30,013 were risk takers and *n* = 87,742 were non-risk takers. The sex distribution and demographics of this data set were comparable with those included in the discovery analysis based on the first genetic release (Supplementary Table 1).

**Table 1:** Description of UK Biobank participants included in the discovery risk-taking GWAS, replication and PRS analyses

	Discovery (1000 genomes)		Replication (HRC)		PRS (HRC)	
	Not risk takers	Risk takers^a^	Not risk takers	Risk takers^a^	Not risk takers	Risk takers^a^
*N*	86,552	29,703	104,263	35,210	104,533	35,198
*N* men	36,679 (0.42)	18,554 (0.63)	41,988 (0.40)	21,453 (0.61)	42,161 (0.40)	21,427 (0.61)
Age (years)	57.2 (7.8)	56.1 (8.1)	57.2 (7.9)	55.9 (8.2)	57.3 (7.9)	56.0 (8.2)
BMI (kg/m^2^)	27.4 (4.9)	27.9 (4.7)	27.2 (4.7)	27.7 (4.7)	27.2 (4.7)	27.7 (4.6)
Current smoker	28,575 (0.33)	11,123 (0.38)	35,804 (0.34)	13,568 (0.39)	36,219 (0.35)	13,684 (0.39)
Ever smoker	37,782 (0.44)	16,052 (0.54)	43,929 (0.42)	18,265 (0.52)	44,316 (0.43)	18,221 (0.52)
Age completed education^b^	16.6 (2.1)	16.6 (2.3)	17.0 (2.1)	16.7 (2.4)	16.6 (2.1)	16.7 (2.4)
Has a degree	24,442 (0.29)	10,235 (0.35)	30,456 (0.29)	12,830 (0.37)	30,672 (0.30)	12,731 (0.36)
Townsend deprivation index	−1.6 (2.9)	−1.3 (3.1)	−1.7 (2.8)	−1.4 (3.0)	−1.7 (2.9)	−1.4 (3.0)
Unstable mood¤	37,429 (0.44)	14,258 (0.49)	44,659 (0.44)	16,852 (0.49)	44,697 (0.44)	16,722 (0.48)
Comparison group^c^	17,024 (0.74)	5418 (0.69)	20,519 (0.74)	6350 (0.69)	20,844 (0.74)	6211 (0.69)
BD*	190 (0.01)	177 (0.02)	215 (0.01)	177 (0.02)	206 (0.01)	189 (0.02)
Single episode depression^c^	1615 (0.08)	519 (0.07)	1860 (0.07)	645 (0.07)	1838 (0.07)	659 (0.07)
Moderate depression^c^	2816 (0.12)	1034 (0.13)	3351 (0.12)	1289 (0.14)	3414 (0.12)	1207 (0.13)
Severe depression^c^	1486 (0.06)	678 (0.08)	1727 (0.06)	797 (0.09)	1733 (0.06)	796 (0.09)
Any depression	5917 (0.26)	2231 (0.29)	6938 (0.25)	2731 (0.29)	6985 (0.25)	2662 (0.29)
Mental health questionnaire	27,494	9479	34,011	11,654	34,060	11,574
BD	330 (0.01)	232 (0.02)	369 (0.01)	290 (0.03)	348 (0.01)	277 (0.02)
MDD	6450 (0.28)	2407 (0.30)	7716 (0.27)	2951 (0.30)	7896 (0.28)	2906 (0.30)
GAD	1893 (0.10)	695 (0.11)	2215 (0.09)	898 (0.11)	2431 (0.10)	831 (0.10)
Any addiction	1491 (0.05)	918 (0.10)	1521 (0.05)	919 (0.08)	1543 (0.05)	955 (0.08)
Alcoholism	569 (0.02)	368 (0.04)	598 (0.02)	381 (0.03)	576 (0.02)	387 (0.03)
Illicit drug addiction	93 (0.003)	101 (0.01)	70 (0.003)	70 (0.01)	78 (0.002)	92 (0.01)
OTC/prescr~t addiction	229 (0.01)	96 (0.01)	22 6 (0.01)	125 (0.01)	239 (0.01)	132 (0.01)
Ever cannabis	4788 (0.17)	2780 (0.29)	6038 (0.18)	3432 (0.29)	5941 (0.17)	3356 (0.29)

*BD* bipolar disorder, *MDD* major depressive disorder, *GAD* general anxiety disorder

^a^Participants who answered “yes” to “do you consider yourself a risk taker?”; for continuous variables, data are presented as mean (standard deviation). For categorical variables, data are presented as n (proportion of group (i.e., risk takers or not risk takers))

^b^Based on a subset of 80,229 subjects; ¤ participants who answered yes to ““Does your mood often go up and down?”

^c^Definitions as per Smith et al.^39^, based on a subset of 29,929 subjects. Addiction phenotypes based upon self-report

### Replication analysis

Approximately half of the participants only present in the second data release were included in the replication analysis, thus after quality control and recommended exclusions, 139,474 white British participants were included. Demographics of the replication sample set are presented in Table 1.

The lead SNPs in the *CADM2* and Chr6 loci were selected for replication. Consistent with the discovery analysis, replication analysis was conducted in PLINK^18^ using logistic regression, assuming a model of additive allelic effects and models were adjusted for sex, age, genotyping array, and the first eight genetic PCs (PCA1–8) to control for hidden population stratification. As two SNP were investigated, *p* < 0.025 was considered significant. Results were meta-analysed using METAL^19^.

### Polygenic risk scores (PRS)

In order to assess the variance explained by the genetic loci identified here, polygenic risk scores (PRSs) were calculated in the remaining 50% of the second genetic data release. Demographics of the PRS sample set are presented in Table 1. After quality control and recommended exclusions, 139,731 white British participants were included in this analysis.

PRS were calculated using *p-*value thresholds of *p* < 1 × 10^−5^, *p* < 0.001, and *p* < 0.05. A score of only GWAS significant SNPs was not conducted, as a 2 SNP score (after linkage disequilibrium (LD)-based pruning) would be underpowered. LD pruning was performed via PLINK on a random sample of 10,000 individuals using an r^2^ > 0.05 in a 250 kb window. The SNP with the lowest *p*-value was selected from each of the LD-clumped SNP sets. Where two or more SNPs from a set had the same *p*-value, the SNP with the larger beta coefficient was used. The scores were calculated in PLINK to produce a per-allele weighted score (without mean imputation). Using STATA, deciles of scores were computed and modelling the effect of the PRS on risk was adjusted for age, sex, chip and PCs 1–8.

### Data mining

SNPs associated (at genome-wide significance) with risk-taking behaviour were further investigated for influence on nearby genes (variant effect predictor, VEP^20^) and for reported associations with relevant traits (GWAS catalogue^21^). Descriptions and known or predicted functions of implicated genes were compiled (GeneCards www.genecards.org and Entrez Gene www.ncbi.nlm.nih.gov/entrez/query.fcgi?db=gene) and global patterns of tissue expression were assessed (GTEx^22^). Exploratory analyses of the impact of significant loci on the expression of nearby genes were carried out using the GTEx Portal “Test your own eQTL” function^22^. In the 13 brain regions available in the GTEx data set, we tested for associations between rs13084531 and *CADM2* expression, and between rs9379971 and the expression of *POM121L2, PRSS16*, *ZNF204P* and *VN1R10P*.

### SNP heritability and genetic correlation analyses

LD score regression (LDSR)^23^ was applied to the GWAS summary statistics to estimate the risk-taking SNP heritability (*h*^2^_SNP_). LDSR was also used to assess genetic correlations between risk-taking behaviour and relevant psychiatric, cognitive and behavioural traits, namely: ADHD, SCZ, BD, MDD, anxiety, PTSD, smoking status (ever smoked), lifetime cannabis use, fluid intelligence, years of education, obesity, and alcohol use disorder.

The importance of the brain in regulation of obesity has been demonstrated^24^, with reward circuits being implicated. The prevalence of obesity in psychiatric illness and the possibility of over-eating being a problem behaviour suggest that there might be a connection between obesity and risk-taking behaviour. Thus, two measures of obesity were included: body mass index (BMI) as a measure of total obesity^24^ and waist-to-hip ratio adjusted for BMI (WHRadjBMI), reflecting metabolically detrimental central obesity^25^.

For the ADHD, SCZ, BD, MDD, anxiety, PTSD, and smoking status, we used GWAS summary statistics provided by the Psychiatric Genomics Consortium (http://www.med.unc.edu/pgc/)^26–32^. For the two obesity phenotypes, GWAS summary statistics for BMI^24^ and WHRadjBMI^25^ were taken from the consortium for the Genetic Investigation of Anthropometric Traits (http://portals.broadinstitute.org/collaboration/giant). Summary statistics for years of education^33^ and fluid intelligence^34^ were downloaded as instructed in the respective publications. Summary statistics for the GWAS of lifetime cannabis use were provided by the International Cannabis Consortium^35^. Summary statistics for GWAS of alcohol consumption^36^ and brain structure volumes^37^ were provided by the authors. Alcohol use disorder was defined using DSM-5 criteria^38^. For this phenotype, a GWAS meta-analysis on genotypes imputed to 1000 Genomes was run with five data sets: COGEND, COGEND2, COGEND-23andMe, COGA, and FSCD. In total, there were *N* = 2983 cases with alcohol use disorder and *N* = 1169 controls. Descriptions of the data sets are in the Supplementary information.

## Results

### Demographic characteristics

A subset of 20,335 participants had repeated assessment of risk-taking behaviour. Reproducibility was good, with consistent responses in 81% of all participants (inconsistent 13%, missing 6%, Supplementary Table 2). Participants with probable mood disorders^39,40^ showed comparable reproducibility compared with those without (consistent 80% vs 82%, inconsistent 15% vs 12%, missing 5% vs 5%, respectively).

For all analyses (discovery, replication, and PRS), small but consistent differences were observed between controls and risk takers with regard to age and BMI (Table 1), but striking differences were observed for sex distribution, smoking, and history of mood disorders: risk takers (compared with non-risk takers) were more often men, more likely to be current or ever-smokers and more likely to suffer from depression, report an addiction or to have used cannabis. Risk takers were also more likely to have a university/college degree.

### GWAS of risk-taking behaviour

**Fig. 1:**
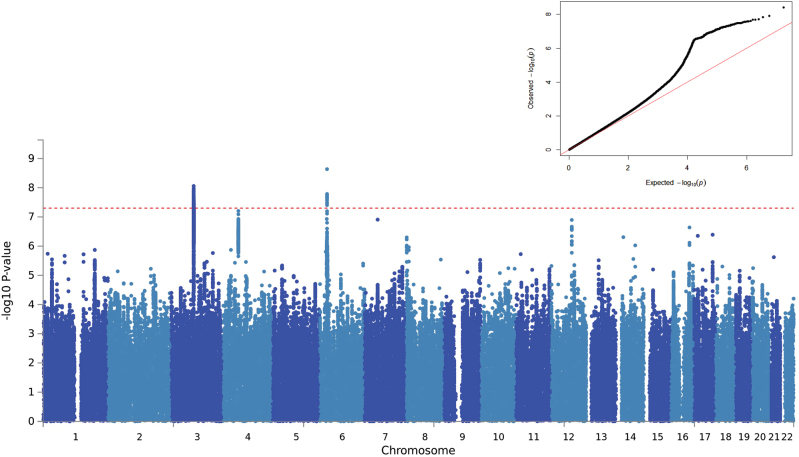
Results of a genome-wide association study of self-reported risk-taking behaviour (1000 Genome imputation). SNPs are plotted along the *X* axis by chromosome and position, with strength of association with self-reported risk-taking behaviour plotted on the *Y* axis. The red line indicates the threshold for GWAS significance (*p* ≤ 5e^−8^). Inset: QQ plot demonstrates deviation from null expectation (solid red line) of the GWAS results (black data points)

GWAS results for risk taking are summarised in Fig. 1 (Manhattan plot), Fig. 1 inset (QQ plot) and Supplementary Table 3. The GWAS data test statistics showed modest deviation from the null (λ_GC_ = 1.13). Considering the sample size, the deviation was negligible (λ_GC_ 1000 = 1.002). LDSR suggested that deviation from the null was due to a polygenic architecture in which *h*^2^_SNP_ accounted for approximately 4% of the population variance in risk-taking behaviour (observed scale *h*^2^_SNP_ = 0.058 (SE 0.006)), rather than inflation due to unconstrained population structure (LD regression intercept = 1.003 (SE 0.008)).

**Fig. 2:**
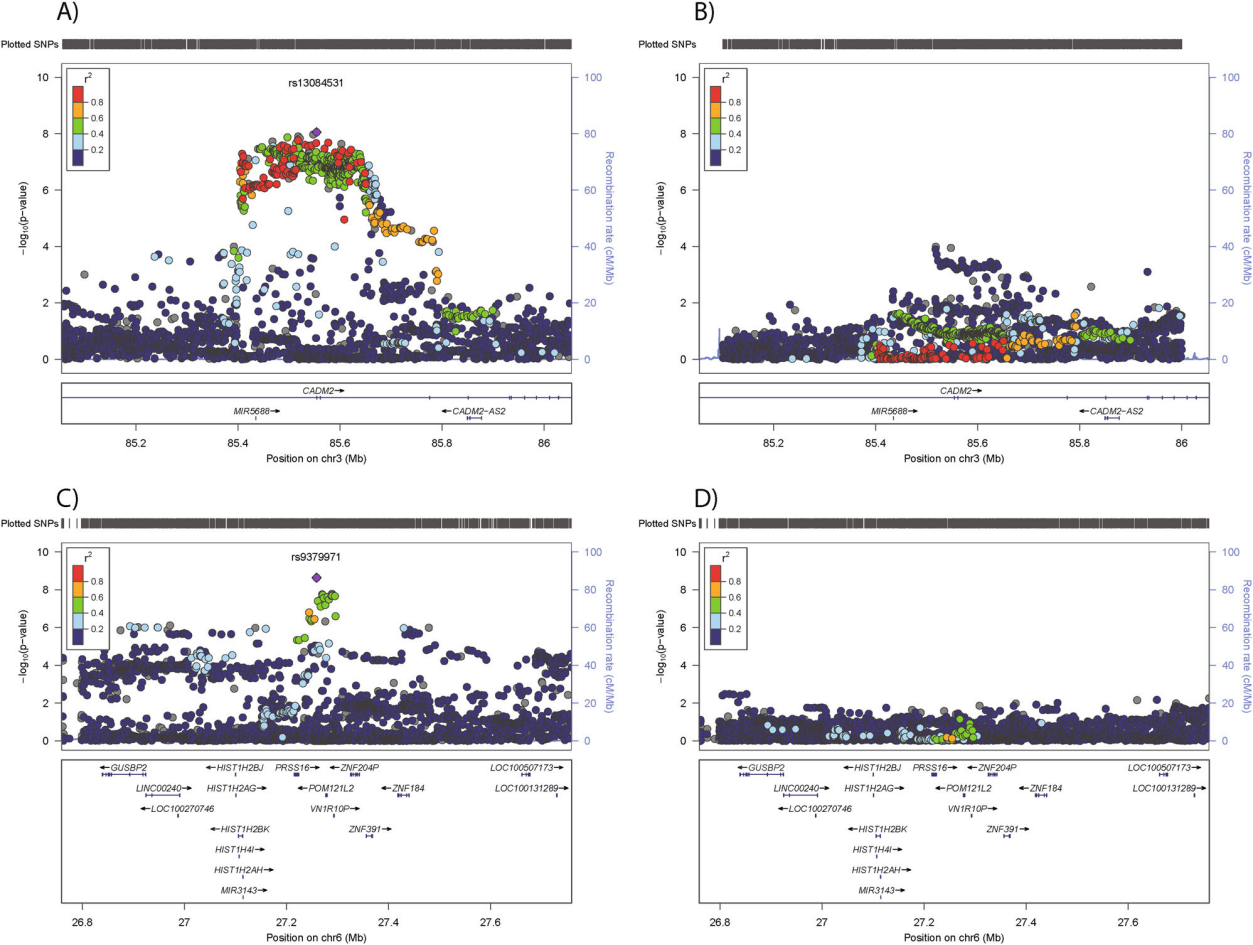
Regional plots for risk-taking-associated loci. **a** Chr3 main analysis results; **b** results of analysis conditioned on Chr3 rs13084531; **c** Chr6 main analysis results; **d** results of analysis conditioned on Chr6 rs9379971. The index SNP is shown as a purple diamond

Two loci were associated with risk-taking behaviour at genome-wide significance, on chromosome 3 and chromosome 6 (Fig. 1 and Supplementary Table 3). The index SNP on chromosome (chr) 3, rs13084531, lies within the *CADM2* gene, however, LD suggests that the signal also encompasses *miR5688*, and borders a *CADM2* anti-sense transcript (*CADM2-AS2*, Fig. 2a). The minor allele of rs13084531 was associated with increased risk-taking (G allele, MAF 0.23, odds ratio (OR) 1.07, confidence interval (CI) 1.04–1.09, *p* 8.75 × 10^–9^). Conditional analysis of the chr3 locus (including rs13084531 as a covariate) is suggestive of a second signal (index SNP rs62250716, MAF 0.36, OR 0.96, CI 0.94–0.98, *p* 8.53 × 10^–5^, LD r^2^ = 0.16 with rs13084531, Fig. 2b and Supplementary Table 3). The LD structure across the chr3 locus supports the possibility of two distinct signals (Supplementary Figure 1).

The chr6 locus lies within the gene-rich human leukocyte antigen (HLA) region (Fig. 2c), where index SNP rs9379971 demonstrated an association between the minor allele and decreased risk-taking (A allele, MAF 0.35, OR 0.95, CI 0.93–0.97, *p* 2.31 × 10^–9^). Conditional analysis (including rs9379971 as a covariate) and assessment of the LD structure across this locus indicated that the associated region probably includes only one signal (Fig. 2d, Supplementary Table 3 and Supplementary Figure 2).

Rerunning the GWAS with the second genetic data release (Supplementary Figure 3) gave similar results, with a modest deviation from the null (λ_GC_ = 1.10, adjusted for sample size λ_GC_ 1000 = 1.002). Consistent with the 1000 Genomes analysis, LDSR suggested that deviation from the null was due to a polygenic architecture with *h*^2^_SNP_ accounting for approximately 5% of the population variance in risk-taking behaviour (observed scale *h*^2^_SNP_ = 0.055 (SE 0.006)). The same *CADM2* locus was GWAS significant (rs62250713, beta 0.0614, SE 0.01, *p* = 8.289 × 10^–10^, minor allele A, MAF 0.36) but the locus on chromosome 6 did not meet the threshold for significance.

### Replication analysis

Both the *CADM2* and chr6 loci demonstrated significant (*p* < 0.025) associations with risk-taking behaviour in the replication analyses (Supplementary Table 4). The *CADM2* locus demonstrated effect sizes comparable with those for the discovery analysis (rs13084531 beta 0.067 for discovery and beta 0.054 SE 0.011 replication). In contrast, the Chr6 locus demonstrated two- to sixfold weaker effects (rs9379971, discovery beta −0.063, replication beta −0.010). The *CADM2* locus met the threshold for GWAS significance in the meta-analysis (Supplementary Table 4) but the Chr6 locus did not. The significant *p*-value for heterogeneity suggests that this association is a false-positive finding.

### PRS analysis

The PRS were significant predictors of risk-taking behaviour, at all *p* thresholds and the variance explained by the model including the PRS was between 0.034 (PRS *p* < 1 × 10-5) and 0.037 (PRS *p* < 0.05) (Supplementary Table 5).

### Data mining

As with the majority of SNPs identified by GWAS, the genome-wide significant SNPs in both loci are non-coding. Current prediction models ascribe only non-coding modifier functions to the 81 genome-wide significant SNPs (VEP^20^, Supplementary Table 6). Expression quantitative trait analysis directly tests association of the index SNPs with expression of nearby transcripts. The chr3 index SNP (rs13084531) lies within the *CADM2* gene and adjacent to a micro RNA, miR5688, and *CADM2-AS2* (Fig. 2 and Supplementary Table 7). Currently, most miRs are predicted (but not reliably proven) to influence transcription of hundreds or thousands of genes. Furthermore, analysing transcription levels of miRs is challenging. Similarly, the importance of anti-sense transcripts such as *CADM2-AS2* is unclear and difficult to assess. *CADM2*, which encodes cell adhesion molecule 2 (also known as synaptic cell adhesion molecule, SynCAM2*)*, is a plausible target gene as it is predominantly expressed in the brain (Supplementary Figure 4A). The risk allele at rs13084531 was associated with increased *CADM2* mRNA levels in several regions of the brain (including the caudate basal ganglia and putamen basal ganglia, hippocampus and hypothalamus, Supplementary Figure 5). *CADM1*, a related cell adhesion molecule, demonstrates overlapping and co-regulated (albeit inversely) expression patterns^41^. It is worth noting that *CADM1* shows a similar, albeit less brain-specific, expression pattern (Supplementary Figure 4B) and that genetic deletion of *Cadm1* in mice results in behavioural abnormalities, including anxiety^42^.

Excitement-seeking is a behavioural trait closely related to risk-taking behaviour^43^, however, the locus reported for excitement seeking was nonsignificant in this study (Chr2, rs11126769, LD R^2^ with the reported rs7600563 = 0.862, major T allele, Beta 0.016, SE 0.011, *p* = 0.1167). Other potentially problematic behaviours, which can be related to risk-taking propensity, have identified the *CADM2* locus (Supplementary Table 8): a recent GWAS of alcohol consumption^44^ identified a significant signal in the *CADM2* locus, where the G allele of rs9841829 was associated with increased alcohol consumption. The same SNP demonstrates genome-wide significance with increased risk-taking behaviour in this study (G, Beta 0.0635, SE 0.012 *p* = 3.34 × 10^−8^, Supplementary Table 3), whereas conditional analysis (Supplementary Table 3) indicates that the signal for alcohol consumption and risk-taking is the same. A GWAS of lifetime cannabis use also highlighted the *CADM2* locus (gene-based rather than SNP-based)^35^. Cognitive function plays a role in traits such as risk-taking, therefore it is worth noting that a GWAS of executive functioning and information processing speed in non-demented older adults from the CHARGE (Cohorts for Heart and Aging Research in Genomic Epidemiology) consortium found that genetic variation in the *CADM2* gene was associated with individual differences in information processing speed^45^. The allele of rs17518584 (LD r^2^ = 0.45 with rs13084531, LD r^2^ = 0.34 with rs62250716) associated with increased processing speed was associated with reduced (self-reported) risk-taking in the current study (Supplementary Table 8, *p* = 1.17 × 10^−7^). Furthermore, a GWAS of educational attainment in the UK Biobank cohort demonstrated a significant signal in *CADM2*^46^. The effect allele of rs56262138 (LD r^2^ = 0.00 with rs13084531, LD r^2^ = 0.00 with rs62250716) for increased educational attainment showed a negative effect on risk-taking behaviour (Supplementary Table 8, *p* = 0.0210).

Day et al. reported an association between the *CADM2* locus and age of reproductive onset in UK Biobank. In a secondary analysis, they also report an association between the same locus, *CADM2*, and risk-taking behaviour (the same phenotype as was used here). However, differences in quality control procedures mean that the lead SNP reported by Day et al. was not available in our analysis. During the revision of this paper, Boutwell et al.^10^ replicated the association between the *CADM2* locus and a number of personality traits including risk-taking (“do you feel comfortable or uncomfortable with taking risks?”), in an independent data set (*n* ~ 140,000).

The *CADM2* locus has also been tentatively associated with longevity^47^ ((Supplementary Table 8) rs9841144, LD r^2^ = 0.99 with rs13084531, LD r^2^ = 0.16 with rs62250716), but associations between *CADM2* SNPs and longevity, survival and attaining 100 years of age in that study were inconsistent, limiting the interpretation of these signals in the context of risk-taking behaviour.

### Genetic correlations

Looking up the risk-taking SNPs in the GWAS results of psychiatric conditions demonstrated little or no effect of the CADM2 SNPs in ADHD, SCZ, PTSD, BPD, or MDD (Supplementary Table 9). In contrast, when considering the entire genome, we found significant positive genetic correlations between the risk-taking phenotype and ADHD (r_g_ = 0.31, SE = 0.13, *p* = 0.01), SCZ (r_g_ = 0.27, SE = 0.04, *p* = 4.54 × 10^−11^), BD (r_g_ = 0.26, SE = 0.07, *p* = 1.73 × 10^−4^), PTSD (r_g_ = 0.51, SE = 0.17, *p* = 0.0018), lifetime cannabis use (r_g_ = 0.41, SE = 0.11, *p* = 0.0001), and smoking (r_g_ = 0.17, SE = 0.07, *p* = 0.01) and a negative genetic correlation with fluid intelligence (r_g_ = −0.15, SE = 0.05, *p* = 0.0013, Table 2). We found no significant genetic correlation between risk-taking and MDD, anxiety, or years of education (Table 2). There was also a significant genetic correlation between risk-taking and BMI (r_g_ = 0.10, SE = 0.03, *p* = 0.003), but a similar correlation was not found for WHRadjBMI. The nonsignificant genetic correlation with alcohol use disorder was interesting because of the strength of the coefficient (r_g_ = 0.22, SE0.31, *p* = 0.47), however, was likely underpowered due to the modest size of the GWAS (*n* = 4 171) and we draw no conclusions about this correlation.

**Table 2:** Genetic correlation between risk-taking and traits relevant to psychiatric disorders

Phenotype	rg	SE	*p*
ADHD	0.378	0.054	**1.80** × **10**^**−12**^
SCZ	0.265	0.040	**4.54** × **10**^**−11**^
BD	0.261	0.070	**1.73** × ^**10−4**^
MDD	0.069	0.084	0.4120
PTSD	0.513	0.165	**0.0018**
Anxiety (case control)	−0.090	0.132	0.4963
Anxiety (quantitative)	−0.123	0.156	0.4289
Ever smoker	0.174	0.068	**0.0102**
Alcohol (heavy vs light)	0.249	0.234	0.2873
Alcohol (quantitative)	0.248	0.082	**0.0026**
Alcohol use disorder	0.221	0.306	0.4700
Lifetime cannabis use	0.406	0.107	**1.00** × **10**^**−4**^
Caudate volume	0.049	0.078	0.5268
Accumbens volumes	0.195	0.143	0.1710
Fluid intelligence	−0.151	0.047	**0.0013**
Years of education	−0.023	0.033	0.4873
BMI	0.102	0.034	**0.0028**
WHRadjBMI	0.087	0.047	0.0655

Bold indicates significant *p* values

*MDD* major depressive disorder, *BD* bipolar disorder; *SCZ* schizophrenia, *ADHD* attention-deficit hyperactivity disorder, *BMI* body mass index, *WHRadjBMI* waist:hip ratio adjusted for BMI. Alcohol dependence DSM-5 criteria: *rg*, regression coefficient, *se*, standard error of the regression coefficient

*p*, *p*-value for the regression analysis

## Discussion

There is a growing emphasis on the importance of using phenotypic traits, which cut across traditional diagnostic groups to investigate the biological basis of psychiatric disorders. Risk-taking behaviour is one such trans-nosological characteristic, recognised clinically as a feature of several disorders, including ADHD, SCZ, and BD. In this study, we identified two loci, on 3p12.1 and 6p22.1, which were associated with self-reported risk-taking behaviour. Replication in an independent set of samples and meta-analysis confirmed the association between risk-taking behaviour and the *CADM2* locus on Chr3 but not the Chr6 locus. The PRS were significant predictors of risk-taking behaviour in a further independent sample set.

The chr6 locus falls within the HLA region, which encodes a large number of genes and is extremely complicated genetically. The false-positive association detected could be because the first data release were selected based on (extremes of) lung function measurements^48^. Considering the potential inflammatory component of lung function and the role of the HLA region in inflammatory responses, it is perhaps not surprising that the discovery analysis demonstrated stronger effect sizes for this locus than the randomly selected general population samples included in the replication analysis.

A key finding of our study was the positive association between Chr3 SNP, rs13084531, and risk-taking behaviour, as well as *CADM2* expression levels. Here, the allele associated with increased self-reported risk-taking behaviour was also associated with increased *CADM2* expression. It is of interest that lack of *Cadm1* in mice was associated with anxiety-related behaviour^42^ and that both *CADM1* and *CADM2* were identified as BMI-associated loci^24^ suggesting that *CADM2* and related family members may be involved in balancing appetitive and avoidant behaviours.

Day and colleagues recently identified 38 genome-wide significant loci for age at first sexual intercourse within the UK Biobank cohort^2^ and two of these loci were within the 3p12.1 region, close to *CADM2* (rs12714592 and rs57401290). The association between rs57401290 (and SNPs in LD) and age at first sexual intercourse was also observed for a number of behavioural traits, including number of sexual partners, number of children, and risk-taking propensity (the same phenotype as was used in this study). In addition, *CADM2* also showed association with information processing speed^45^ and educational attainment^46^, highlighting the complexity of relationships between cognitive performance and risk taking. Taken together, this evidence suggests that *CADM2* plays a fundamental role in risk-taking behaviours, and may be a gene involved in the nexus of cognitive and reward-related processes that underlie them.

A perhaps surprising observation was the increased frequency of having a university degree in self-reported risk takers, compared with controls, despite the negative (albeit nonsignificant) association between years of education and risk-taking behaviour. It is important to note that risk-taking behaviour includes a number of different aspects, including delayed gratification, assessment of positive and negative consequences of risk, impulse control, reward signalling. It is possible that risk-taking behaviour assessed in a clinical mental health setting could reflect a different aspect of these processes compared with self-reported risk-taking behaviour. Risk-taking behaviour assessed in a clinical mental health setting might demonstrate significantly different associations with education, compared with self-reported risk-taking behaviour. These observations underscore the complexity between risk-taking and educational attainment, and highlight differences between genetic and phenotypic relationships. They may also be indicative of selection bias within the UK Biobank cohort towards more highly educated individuals.

Another key finding was genetic correlation between self-reported risk taking and obesity. Although there are likely to be a range of potential mechanisms linking risk-taking behaviour with obesity, evidence of a shared genetic component is in keeping with work that has highlighted the importance of the central nervous system in the regulation of obesity (BMI), particularly brain regions involved in cognition, learning, and reward^24^. In contrast, central fat accumulation (WHRadjBMI) is primarily regulated by adipose tissue^25^, which fits with the lower, nonsignificant genetic correlation between risk-taking behaviour and this measure. Two SNPs (rs13078807 and rs13078960) in the *CADM2* locus have previously been associated with BMI^24,49,50^, but while these SNPs tag each other (LD r^2^ = 0.99), the LD between the risk-taking index SNP or possible secondary signal is low (LD r^2^ = 0.31 and 0.01 for rs13084531 and rs62250716, respectively), suggesting that these are distinct signals.

It is perhaps unsurprising that we identified genetic correlations between risk taking and smoking. Similarly, risk taking and impulsive behaviour is a core feature of ADHD and BD, suggesting substantial genetic overlap between variants predisposing to risk-taking behaviour and these disorders. The genetic correlation between risk taking and SCZ is of interest because SCZ is commonly comorbid with substance abuse disorders^51^. The correlation between risk taking and PTSD is perhaps plausible if we accept that risk takers may be more likely to find themselves in high-risk situations with the potential to cause psychological trauma. Overall, these correlations suggest that studying dimensional traits such as risk-taking has the potential to inform the biology of complex psychiatric disorders.

### Strengths and limitations

We acknowledge that Day et al. have previously reported an association for risk taking within the *CADM2* locus. Strengths of our study include the use of a more conservative and standardised methodology and reporting of results across the entire genome. A risk-taking locus was identified in the *CADM2* locus and we have shown that *CADM2* may contain a second signal. Furthermore, we have investigated the possibility of a sex-specific effect of these loci, provided evidence highlighting possible candidate genes at both loci and confirmed the importance of this phenotype in relation to psychiatric illness. In short, our report provides a fuller understanding of the genetic basis of risk-taking behaviour. Despite this, we highlight some limitations. The risk-taking phenotype used was a self-reported measure, based on response to a single question, and is therefore open to responder bias. It is also plausible that there are distinct subtypes of risk-taking behaviour (for example disinhibition, sensation seeking, and calculated risks). Whether the single question used in our analyses captures all, or only some, of these is not clear. Having identified genetic loci associated with other traits related to risk taking and other problem behaviours (such as alcohol consumption and cannabis use) provides added support for the validity of this phenotype. It would be of interest to investigate whether the loci identified here are also associated with more quantitative and objective measures of risk taking; however, such measures were not available in the UK Biobank data set.

## Conclusion

In summary, we have identified a polygenic basis for self-reported risk-taking behaviour and the *CADM2* locus, which contains variants likely to play a role in predisposition to this complex but important phenotype. The identification of significant genetic correlations between risk taking and several psychiatric disorders, as well as with smoking and obesity, suggest that future work on this trait may clarify mechanisms underlying several common psychopathological and physical health conditions, which are important for public health and well-being.

## Electronic supplementary material

Supplemental info

Supplemental Methods

Supplemental Table 1

Supplemental Table 2

Supplemental Table 3

Supplemental Table 4

Supplemental Table 5

Supplemental Table 6

Supplemental Table 7

Supplemental Table 8

Supplemental Table 9

Supplemental Figure 1

Supplemental Figure 2

Supplemental Figure 3

Supplemental Figure 4

Supplemental Figure 5

## References

[1] Schoenfelder EN, Kollins SH (2016). Topical review: ADHD and health-risk behaviors: toward prevention and health promotion. J. Pediatr. Psychol..

[2] Day FR (2016). Physical and neurobehavioral determinants of reproductive onset and success. Nat. Genet..

[3] Reinharth J, Braga R, Serper M (2017). Characterization of risk-taking in adults with bipolar spectrum disorders. J. Nerv. Ment. Dis..

[4] de Haan L, Egberts AC, Heerdink ER (2015). The relation between risk-taking behavior and alcohol use in young adults is different for men and women. Drug. Alcohol Depend..

[5] Kreek MJ, Nielsen DA, Butelman ER, LaForge KS (2005). Genetic influences on impulsivity, risk taking, stress responsivity and vulnerability to drug abuse and addiction. Nat. Neurosci..

[6] Kornreich C (2017). Conditional reasoning in schizophrenic patients. Evol. Psychol..

[7] Cheng GL, Tang JC, Li FW, Lau EY, Lee TM (2012). Schizophrenia and risk-taking: impaired reward but preserved punishment processing. Schizophr. Res..

[8] Cope EC, Gould E (2017). New evidence linking obesity and food addiction. Biol. Psychiatry.

[9] Cuthbert BN, Insel TR (2013). Toward the future of psychiatric diagnosis: the seven pillars of RDoC. BMC Med..

[10] Boutwell B (2017). Replication and characterization of CADM2 and MSRA genes on human behavior. Heliyon.

[11] Reddy LF (2014). Impulsivity and risk taking in bipolar disorder and schizophrenia. Neuropsychopharmacology.

[12] Sudlow C (2015). UK biobank: an open access resource for identifying the causes of a wide range of complex diseases of middle and old age. PLoS. Med..

[13] UK Biobank. Genotype imputation and genetic association studies of UK Biobank, Interim Data Release (2015)..

[14] Delaneau O, Zagury JF, Marchini J (2013). Improved whole-chromosome phasing for disease and population genetic studies. Nat. Methods.

[15] Howie B, Marchini J, Stephens M (2011). Genotype imputation with thousands of genomes. G3 (Bethesda).

[16] Huang J (2015). Improved imputation of low-frequency and rare variants using the UK10K haplotype reference panel. Nat. Commun..

[17] UK Biobank. Genotyping of 500,000 UK Biobank participants. Description of sample processing workflow and preparation of DNA for genotyping. https://biobank.ctsu.ox.ac.uk/crystal/docs/genotyping_sample_workflow.pdf (2015).

[18] Purcell S (2007). PLINK: a tool set for whole-genome association and population-based linkage analyses. Am. J. Hum. Genet..

[19] Willer CJ, Li Y, Abecasis GR (2010). METAL: fast and efficient meta-analysis of genomewide association scans. Bioinformatics.

[20] McLaren W (2016). The Ensembl variant effect predictor. Genome Biol..

[21] MacArthur J (2017). The new NHGRI-EBI catalog of published genome-wide association studies (GWAS catalog). Nucleic Acids Res..

[22] GTEx Consortium. The genotype-tissue expression (GTEx) project. *Nat. Genet.* **45**, 580–585 (2013). 10.1038/ng.2653 PMC4010069 23715323

[23] Bulik-Sullivan B (2015). An atlas of genetic correlations across human diseases and traits. Nat. Genet..

[24] Locke AE (2015). Genetic studies of body mass index yield new insights for obesity biology. Nature.

[25] Shungin D (2015). New genetic loci link adipose and insulin biology to body fat distribution. Nature.

[26] Schizophrenia Working Group of the Psychiatric Genetics Consortium. Genome-wide association study identifies five new schizophrenia loci. *Nat. Genet.* **43**, 969–976 (2011). 10.1038/ng.940 PMC3303194 21926974

[27] Ruderfer DM, Fanous AH, Ripke S, McQuillin A, Amdur RL (2014). Schizophrenia Working Group of Psychiatric Genomics C et al. Polygenic dissection of diagnosis and clinical dimensions of bipolar disorder and schizophrenia. Mol. Psychiatry.

[28] Major Depressive Disorder Working Group of the Psychiatric Genetics Consortium, Ripke, S. et al. A mega-analysis of genome-wide association studies for major depressivedisorder. *Mol. Psychiatry* **18**, 497–511 (2013). 10.1038/mp.2012.21 PMC3837431 22472876

[29] Neale BM (2010). Meta-analysis of genome-wide association studies of attention-deficit/hyperactivity disorder. J. Am. Acad. Child. Adolesc. Psychiatry.

[30] Tabacco and Genetics Consortium. Genome-wide meta-analyses identify multiple loci associated with smoking behavior. *Nat. Genet.* **42**, 441–447 (2010). 10.1038/ng.571 PMC2914600 20418890

[31] Duncan, L. E. et al. Largest GWAS of PTSD (N = 20 070) yields genetic overlap with schizophrenia and sex differences in heritability. *Mol. Psychiatry*10.1038/mp.2017.77 (2017).. 10.1038/mp.2017.77 PMC5696105 28439101

[32] Otowa T (2016). Meta-analysis of genome-wide association studies of anxiety disorders. Mol. Psychiatry.

[33] Okbay A (2016). Genome-wide association study identifies 74 loci associated with educational attainment. Nature.

[34] Sniekers S (2017). Genome-wide association meta-analysis of 78,308 individuals identifies new loci and genes influencing human intelligence. Nat. Genet.

[35] Stringer S (2016). Genome-wide association study of lifetime cannabis use based on a large meta-analytic sample of 32 330 subjects from the International Cannabis Consortium. Transl. Psychiatry.

[36] Schumann G (2016). KLB is associated with alcohol drinking, and its gene product beta-Klotho is necessary for FGF21 regulation of alcohol preference. Proc. Natl. Acad. Sci. USA.

[37] Hibar DP (2015). Common genetic variants influence human subcortical brain structures. Nature.

[38] American Psychiatric Association. *Diagnostic and Statistical Manual of Mental Disorders, Fifth Edition, DSM-5*. (American Psychiatric Association: Arlington, VA, 2013).

[39] Smith DJ (2013). Prevalence and characteristics of probable major depression and bipolar disorder within UK biobank: cross-sectional study of 172,751 participants. PLoS. ONE.

[40] Davis K. A. S. et al. Mental Health in UK Biobank—development, implementation and results from an online questionnaire completed by 157,366 participants. *BJPsych. Open* (in press). 10.1192/bjo.2018.12 PMC6020276 29971151

[41] Pietri T, Easley-Neal C, Wilson C, Washbourne P (2008). Six cadm/SynCAM genes are expressed in the nervous system of developing zebrafish. Dev. Dyn..

[42] Tanabe Y (2013). Synaptic adhesion molecules in Cadm family at the neuromuscular junction. Cell. Biol. Int..

[43] Terracciano A (2011). Meta-analysis of genome-wide association studies identifies common variants in CTNNA2 associated with excitement-seeking. Transl. Psychiatry.

[44] Toni-Kim Clarke, Mark J et al. Genome-wide association study of alcohol consumption and genetic overlap with other health-related traits in UK Biobank (N = 112,117). 2017. 10.1038/mp.2017.153 PMC5622124 28937693

[45] Ibrahim-Verbaas CA (2016). GWAS for executive function and processing speed suggests involvement of the CADM2 gene. Mol. Psychiatry.

[46] Davies G (2016). Genome-wide association study of cognitive functions and educational attainment in UK Biobank (N = 112 151). Mol. Psychiatry.

[47] Broer L (2015). GWAS of longevity in CHARGE consortium confirms APOE and FOXO3 candidacy. J. Gerontol. A Biol. Sci. Med. Sci..

[48] Wain LV (2017). Genome-wide association analyses for lung function and chronic obstructive pulmonary disease identify new loci and potential druggable targets. Nat. Genet..

[49] Berndt SI (2013). Genome-wide meta-analysis identifies 11 new loci for anthropometric traits and provides insights into genetic architecture. Nat. Genet..

[50] Speliotes EK (2010). Association analyses of 249,796 individuals reveal 18 new loci associated with body mass index. Nat. Genet..

[51] Thoma P, Daum I (2013). Comorbid substance use disorder in schizophrenia: a selective overview of neurobiological and cognitive underpinnings. Psychiatry Clin. Neurosci..

