# Supplementary material for: Genome-wide analysis of self-reported risk-taking behaviour and cross-disorder genetic correlations in the UK Biobank cohort
Source: Transl Psychiatry. 2018 Feb 2;8:39. doi: 10.1038/s41398-017-0079-1 (PMC5804026; doi:10.1038/s41398-017-0079-1)
Supplement: Supplementary file 1 — Supplemental info [file 41398_2017_79_MOESM1_ESM.docx]

**Genome-wide analysis of risk-taking behaviour and cross-disorder genetic correlations in 116 255 individuals from the UK Biobank cohort.**

**Supplementary Information contents**

**Supplementary Methods:** Alcohol use disorder cohort descriptions

**Supplementary Figure 1:** LD structure of the chr3 locus.

**Supplementary Figure 2:**  LD structure of the chr6 locus.

**Supplementary Figure 3:**  Manhattan plot of the discovery GWAS using the HRC imputation.

**Supplementary Figure 4:** Genotype-specific gene expression of *CADM2* in the specific brain regions.

**Supplementary Figure 5**: Genotype-specific expression of CADM2 in specific brain regions

**Supplementary Table 1.** Description of UK Biobank participants in the discovery analyses using first (1000Genomes) or second (HPC) data release.

**Supplementary Table 2:** Test-retest numbers and coefficients.

**Supplementary Table 3.** Genome-wide significant loci associated with risk-taking in UK Biobank: basic and conditional analyses

**Supplementary Table 4.** Genome-wide significant loci associated with risk-taking in UK Biobank and replication analyses

**Supplementary Table 5**: Effects of risk-taking PRS on risk-taking behaviour in an independent sample set.

**Supplementary Table 6**: Variant Effect Predictor summary of impact of *CADM2* SNPs on the CADM2 protein

**Supplementary Table 7:** Genes in the *CADM2* locus

**Supplementary Table 8**: Previously reported signals in the *CADM2* locus and results in the risk-taking GWAS

**Supplemental Table 9**: Association of risk-taking loci with psychiatric traits
