# Supplementary material for: Genome-wide analysis of self-reported risk-taking behaviour and cross-disorder genetic correlations in the UK Biobank cohort
Source: Transl Psychiatry. 2018 Feb 2;8:39. doi: 10.1038/s41398-017-0079-1 (PMC5804026; doi:10.1038/s41398-017-0079-1)
Supplement: Supplementary file 2 — Supplemental Methods [file 41398_2017_79_MOESM2_ESM.docx]

**Genome-wide analysis of risk-taking behaviour in 116 255 individuals in UK Biobank and cross-disorder genetic correlation.**

**Supplementary Methods**

**Description of cohorts contributing to the alcohol use disorder meta-analysis (unpublished as of 20170608)**

**COGEND:**

Recruitment Sites: Washington University (St. Louis, MO), Henry Ford Health System (Detroit, MI)

Recruitment Dates: 2002-2007

Instrument: Semi-Structured Assessment of Nicotine Dependence (SSAND)

Inclusion Criteria: age 25-44, English speaking, smoked 100 cigarettes lifetime, current FTND >4 for case, lifetime FTND < 1 for control

The Collaborative Genetic Study of Nicotine Dependence (COGEND) was initiated to detect and characterize genes that alter risk for heavy tobacco consumption, nicotine dependence, and related phenotypes. Community-based recruitment was used to enroll nicotine dependent cases and non-dependent smoking controls in St. Louis, Missouri and Detroit, Michigan between 2002 and 2007. Institutional Review Board approval was obtained at all data collection sites prior to enrolling participants, and all participants provided informed consent. Participants were recruited using the Missouri Family Registry in St. Louis and Health Maintenance Organizations in Detroit. Potential participants were screened to determine eligibility. All participants had to be between the ages of 25-44 years and speak English. Nicotine dependent cases were defined as current smokers with a Fagerström Test for Nicotine Dependence (FTND) score of 4 or greater ^1^. Control status was defined as smoking at least 100 cigarettes lifetime, but never being nicotine dependent (lifetime FTND score < 1). Other substance dependence diagnoses or comorbid disorders were not exclusionary criteria. Those who qualified as a nicotine dependent case or non-dependent control completed an in-person comprehensive interview and donated a blood sample for genetic analysis.

Following the informed consent process, participants were assessed for baseline demographics, psychiatric disorders, and substance use history using a Computer Assisted Personal Interview (CAPI) version of the Semi-Structured Assessment of Nicotine Dependence (SSAND), which was developed specifically for COGEND. The SSAND is modeled after the Semi-Structured Assessment for the Genetics of Alcoholism (SSAGA) ^2, 3^. The SSAGA is a validated instrument developed to provide a detailed evaluation of alcohol, nicotine and other substance use disorders. The SSAGA has been utilized in many large-scale genetic studies of substance dependence ^4, 5^ and has high test-retest reliability in short-term (one week) and long-term (5 year) studies ^6, 7^. Substance use disorder was assessed based on DSM-IV ^8^ and DSM5 ^9^ criteria. Nicotine dependence was also assessed using the Fagerström Test for Nicotine Dependence (FTND) ^1^. Participants also completed CAPI versions of the NEO Five-Factor Inventory ^10^, the Nicotine Dependence Syndrome Scale ^11^, and the Wisconsin Inventory of Smoking Dependence Motives ^12^.

Participants provided a blood sample for genetic analysis.

**COGEND2**

Recruitment Site: Washington University (St. Louis, MO)

Recruitment Dates: 2011-2014

Instrument: Semi-Structured Assessment of Nicotine Dependence-version 2 (SSAND-II)

Inclusion Criteria: age 25-44, English speaking, current FTND >4 for case, lifetime FTND < 1 for control

The Collaborative Genetic Study of Nicotine Dependence (COGEND) was initiated to detect and characterize genes that alter risk for heavy tobacco consumption, nicotine dependence, and related phenotypes. Additional recruitment was undertaken to expand the sample. Institutional Review Board approval was obtained at Washington University prior to enrolling participants, and all participants provided informed consent. Participants were recruited from the St. Louis metropolitan area between 2011 and 2014 via marketing lists, flyers, and street recruitment. Potential participants were screened to determine eligibility. All participants had to be between the ages of 25-44 years and speak English. Nicotine dependent cases were defined as current smokers with a Fagerström Test for Nicotine Dependence (FTND) score of 4 or greater ^1^. Control status was defined as smoking at least 40 cigarettes lifetime, but never being nicotine dependent (lifetime FTND score < 1). Other substance dependence diagnoses or comorbid disorders were not exclusionary criteria. Those who qualified as a nicotine dependent case or non-dependent control completed an in-person comprehensive interview and donated a blood sample for genetic analysis.

Following the informed consent process, participants were assessed for baseline demographics, psychiatric disorders, and substance use history using a modified Computer Assisted Personal Interview (CAPI) version of the Semi-Structured Assessment of Nicotine Dependence (SSAND). The SSAND is modeled after the Semi-Structured Assessment for the Genetics of Alcoholism (SSAGA) ^2, 3^. The SSAGA is a validated instrument developed to provide a detailed evaluation of alcohol, nicotine and other substance use disorders. The SSAGA has been utilized in many large-scale genetic studies of substance ^4, 5^ and has high test-retest reliability in short-term (one week) and long-term (5 year) studies ^6, 7^. Substance use disorder was assessed based on DSM-IV ^8^ and DSM5 ^9^ criteria. Nicotine dependence was also assessed using the Fagerström Test for Nicotine Dependence (FTND) ^1^. Participants also completed CAPI versions of the NEO Five-Factor Inventory ^10^, the Nicotine Dependence Syndrome Scale ^11^, and the Wisconsin Inventory of Smoking Dependence Motives ^12^.

Participants provided a blood sample for genetic analysis.

**COGEND-23andMe**

Recruitment Site: Washington University (St. Louis, MO)

Recruitment Dates: 2014-2015 (recruitment is ongoing)

Instruments: Semi-Structured Assessment of Nicotine Dependence-short version (SSAND-Short) used in 2014 Semi-Structured Assessment of Nicotine Dependence–short version 2015 (SSAND-Short 2015) used in 2015

Inclusion Criteria: age 25-44, English speaking, smoked 100 cigarettes lifetime, exhaled carbon monoxide level > 7 parts per million, smoked > 15 days during the past month

The Collaborative Genetic Study of Nicotine Dependence (COGEND) was initiated to detect and characterize genes that alter risk for heavy tobacco consumption, nicotine dependence, and related phenotypes. We undertook a further extension to study biomarkers of smoking. Institutional Review Board approval was obtained at Washington University prior to enrolling participants, and all participants provided informed consent. Participants were recruited from the St. Louis metropolitan area between 2014 and 2015 via internet advertising, Facebook, flyers, and word of mouth. All participants were current smokers as demonstrated by an exhaled carbon monoxide level > 7 parts per million and self-reported smoking on > 15 days during the past month. Participants were required to have smoked 100 cigarettes lifetime, be between the ages of 25-44 years and speak English. Other substance use or comorbid conditions were not exclusionary criteria. Those who qualified completed an in-person comprehensive interview, provided measures of exhaled carbon monoxide, and donated a saliva sample for genetic analysis.

Following the informed consent process, participants were assessed for baseline demographics and substance use history using a modified Computer Assisted Personal Interview (CAPI) version of the Semi-Structured Assessment of Nicotine Dependence (SSAND). The SSAND is modeled after the Semi-Structured Assessment for the Genetics of Alcoholism (SSAGA) ^2, 3^. The SSAGA is a validated instrument developed to provide a detailed evaluation of alcohol, nicotine and other substance use disorders. The SSAGA has been utilized in many large-scale genetic studies of substance dependence ^4, 5^ and has high test-retest reliability in short-term (one week) and long-term (5 year) studies ^6, 7^. Substance use disorder was assessed based on DSM-IV ^8^and DSM5 ^9^criteria. Nicotine dependence was also assessed using the Fagerström Test for Nicotine Dependence (FTND) ^1^. Healthcare literacy was assessed using the Rapid Estimate of Adult Literacy in Medicine, Revised (REALM-R) ^13^.

Participants provided saliva samples for genetic analysis using 23andMe DNA collection kits. 23andMe is a privately held [personal genomics](http://en.wikipedia.org/wiki/Personal_genomics) and [biotechnology](http://en.wikipedia.org/wiki/Biotechnology) company that produces high quality genetic data in CLIA-certified laboratories. The success rate of genotyping submitted saliva samples was 97%. In addition to data cleaning performed by 23andMe, we performed additional checks including individual sample quality, SNP quality, Hardy-Weinberg Equilibrium (HWE), duplicates, and relatedness across participants. We required at least a 98% call rate across all SNPs for a sample to be included in analyses. At a SNP level, we required at least a 98% call rate for each SNP in the sample. Relatedness across participants was examined to make sure that our participants were independent. Setting thresholds of per sample call rate of 98%, minor allele frequency of 1% or greater, and HWE p value more than 10^-10,^, we had a final SNP set of 488,487 variants with a mean call rate of 99.89% per sample and average 99.79% call rate per SNP, demonstrating the high quality genetic data generated by 23andMe services.

**COGA**

The Collaborative Study on the Genetics of Alcoholism (COGA) was initiated in 1989 and is a large-scale family study that has had as its primary aim the identification of genes that contribute to alcoholism susceptibility and related characteristics ^4^. Subjects were recruited from 7 cities across the U.S. and Institutional Review Boards at all sites approved the protocols. Alcohol dependent probands were recruited from treatment facilities and were required to meet criteria for DSM-III-R Alcohol Dependence ^14^ and Feighner Alcoholism at the Definite level ^15^. A set of comparison families was drawn from the same communities as the families recruited through the alcohol dependent probands. After obtaining informed consent, participants were assessed using a comprehensive personal interview developed for this project, the Semi-Structured Assessment for the Genetics of Alcoholism (SSAGA), which gathers detailed information on alcoholism-related symptoms along with other drugs and psychiatric symptoms ^2, 3^. The SSAGA has high test-retest reliability in short-term (one week) and long-term (5 year) studies ^6, 7^. Families ascertained through an alcohol dependent proband with three or more first-degree relatives who were alcohol dependent were invited for more extensive testing, including neurophysiology evaluations and a battery of neuropsychological assessments. Participants also provided a blood sample for genetic analysis.

From the family study, unrelated cases and controls were selected for association testing.

**FSCD**

The Family Study of Cocaine Dependence (FSCD) was initiated in 2000 with the primary goal of increasing understanding of the familial and non-familial antecedents and consequences of cocaine dependence ^16^. Cocaine dependent subjects were recruited from publicly and privately funded inpatient and outpatient chemical dependency treatment centers in the St. Louis, Missouri region. Eligibility requirements included DSM-IV cocaine dependence ^8^, being at least 18 years of age, speaking fluent English, and having a full sibling within five years of their age who was willing to participate. Community-based comparison subjects were recruited through driver’s license records from the Missouri Family Registry maintained by Washington University in St. Louis for research purposes. Community-based comparison participants were matched to cocaine dependent subjects based on date of birth (within 1 year), ethnicity, gender, and zip code. The Institutional Review Board at Washington University approved the protocol.

Following the informed consent process, participants were assessed for baseline demographics, psychiatric disorders, and substance use history using the Semi-Structured Assessment of Cocaine Dependence, which was developed specifically for this study. The interview is modeled after the Semi-Structured Assessment for the Genetics of Alcoholism (SSAGA) ^2, 3^. The SSAGA is a validated instrument developed to provide a detailed evaluation of alcohol, nicotine and other substance use disorders. The SSAGA has been utilized in many large-scale genetic studies of substance dependence (^4, 5^ and has high test-retest reliability in short-term (one week) and long-term (5 year) studies ^6, 7^. Participants also provided a blood sample for genetic analysis.

**References**

1. Heatherton TF, Kozlowski LT, Frecker RC, Fagerstrom KO. The Fagerstrom Test for Nicotine Dependence: a revision of the Fagerstrom Tolerance Questionnaire. *Br J Addict* 1991; **86**(9)**:** 1119-1127.

2. Bucholz KK, Cadoret R, Cloninger CR, Dinwiddie SH, Hesselbrock VM, Nurnberger JI, Jr. *et al.* A new, semi-structured psychiatric interview for use in genetic linkage studies: a report on the reliability of the SSAGA. *J Stud Alcohol* 1994; **55**(2)**:** 149-158.

3. Hesselbrock M, Easton C, Bucholz KK, Schuckit M, Hesselbrock V. A validity study of the SSAGA--a comparison with the SCAN. *Addiction* 1999; **94**(9)**:** 1361-1370.

4. Begleiter H RT, Hesselbrock V, Porjesz B, Li TK, Schuckit M, Edenberg HJ, Rice. The Collaborative Study on the Genetics of Alcoholism. *Alcohol Health Res World* 1995; **19**(3)**:** 228-236.

5. Gelernter J, Liu X, Hesselbrock V, Page GP, Goddard A, Zhang H. Results of a genomewide linkage scan: support for chromosomes 9 and 11 loci increasing risk for cigarette smoking. *Am J Med Genet B Neuropsychiatr Genet* 2004; **128B**(1)**:** 94-101.

6. Bucholz KK, Hesselbrock VM, Shayka JJ, Nurnberger JI, Jr., Schuckit MA, Schmidt I *et al.* Reliability of individual diagnostic criterion items for psychoactive substance dependence and the impact on diagnosis. *J Stud Alcohol* 1995; **56**(5)**:** 500-505.

7. Culverhouse R, Bucholz KK, Crowe RR, Hesselbrock V, Nurnberger JI, Jr., Porjesz B *et al.* Long-term stability of alcohol and other substance dependence diagnoses and habitual smoking: an evaluation after 5 years. *Arch Gen Psychiatry* 2005; **62**(7)**:** 753-760.

8. American Psychiatric Association. Diagnostic and statistical manual of mental disorders. 4th edn. American Psychiatric Association: Washington DC, 1994.

9. American Psychiatric Association. Diagnostic and Statistical Manual of Mental Disorders: DSM5. American Psychiatric Association: Washington, D.C, 2013.

10. Costa PT Jr MR. Manual for the Revised NEO Personality Inventory (NEO-PIR) and NEO Five-Factor Inventory (NEO-FFI). . Psychological Assessment Resources: Odessa, FL., 1992.

11. Shiffman S, Waters A, Hickcox M. The nicotine dependence syndrome scale: a multidimensional measure of nicotine dependence. *Nicotine Tob Res* 2004; **6**(2)**:** 327-348.

12. Piper ME, Piasecki TM, Federman EB, Bolt DM, Smith SS, Fiore MC *et al.* A multiple motives approach to tobacco dependence: the Wisconsin Inventory of Smoking Dependence Motives (WISDM-68). *J Consult Clin Psychol* 2004; **72**(2)**:** 139-154.

13. Bass PF, 3rd, Wilson JF, Griffith CH. A shortened instrument for literacy screening. *J Gen Intern Med* 2003; **18**(12)**:** 1036-1038.

14. American Psychiatric Association. Diagnostic and Statistical Manual of Mental Disorders (DSM-III-R) American Psychiatric Association Washington, D.C, 1987.

15. Feighner JP, Robins E, Guze SB, Woodruff RA, Jr., Winokur G, Munoz R. Diagnostic criteria for use in psychiatric research. *Arch Gen Psychiatry* 1972; **26**(1)**:** 57-63.

16. Bierut LJ, Strickland JR, Thompson JR, Afful SE, Cottler LB. Drug use and dependence in cocaine dependent subjects, community-based individuals, and their siblings. *Drug Alcohol Depend* 2008; **95**(1-2)**:** 14-22.
