# Supplementary material for: Genome-wide analysis of self-reported risk-taking behaviour and cross-disorder genetic correlations in the UK Biobank cohort
Source: Transl Psychiatry. 2018 Feb 2;8:39. doi: 10.1038/s41398-017-0079-1 (PMC5804026; doi:10.1038/s41398-017-0079-1)
Supplement: Supplementary file 3 — Supplemental Table 1 [file 41398_2017_79_MOESM3_ESM.docx]

| **Supplementary Table 1. Description of UK Biobank participants in the discovery analyses using first (1000Genomes) or second (HPC) data release.** | | | | | |  |
| --- | --- | --- | --- | --- | --- | --- |
|  | Discovery (100Genomes) | | | Discovery (HPC) | | |
|  | Not risk takers | Risk takers‡ | Not risk takers | | Risk takers‡ |  |
| N | 86 552 | 29 703 | 87742 | | 30013 |  |
| N men | 36 679 (0.42) | 18 554 (0.63) | 37 200 (0.42) | | 18 758 (0.63) |  |
| Age (years) | 57.2 (7.8) | 56.1 (8.1) | 57.2 (7.8) | | 56.0 (8.1) |  |
| BMI (kg/m2) | 27.4 (4.9) | 27.9 (4.7) | 27.4 (4.9) | | 27.9 (4.7) |  |
| Current smoker | 28 575 (0.33) | 11 123 (0.38) | 28 950 (0.33) | | 11 246 (0.38) |  |
| Ever smoker | 37 782 (0.44) | 16 052 (0.54) | 38 289 (0.44) | | 16 209 (0.54) |  |
| Age completed education# | 16.6 (2.1) | 16.6 (2.3) | 16.6 (4.9) | | 16.7 (2.4) |  |
| Has a degree | 24 442 (0.29) | 10 235 (0.35) | 24 859 (0.29) | | 10 356 (0.35) |  |
| Townsend deprivation index | -1.6 (2.9) | -1.3 (3.1) | -2.0 (2.9) | | -1.3 (3.1) |  |
| Unstable mood¤ | 37 429 (0.44) | 14 258 (0.49) | 37 902 (0.43) | | 14 367 (0.48) |  |
| Comparison group* | 17 024 (0.74) | 5 418 (0.69) | 17 282 (0.74) | | 5 515 (0.69) |  |
| BD* | 190 (0.01) | 177 (0.02) | 200 (0.01) | | 175 (0.02) |  |
| single episode depression* | 1 615 (0.08) | 519 (0.07) | 1635 (0.07) | | 540 (0.07) |  |
| Moderate depression* | 2 816 (0.12) | 1 034 (0.13) | 2885 (0.12) | | 1 061 (0.13) |  |
| Severe depression* | 1 486 (0.06) | 678 (0.08) | 15 008 (0.06) | | 674 (0.08) |  |
| any depression | 5 917 (0.26) | 2 231 (0.29) | 5 998 (0.26) | | 2 275 (0.29) |  |
| Mental Health Questionnaire | 27 494 | 9 479 | 27 799 | | 9 640 |  |
| BD | 330 (0.01) | 232 (0.02) | 327 (0.01) | | 236 (0.02) |  |
| MDD | 6 450 (0.28) | 2 407 (0.30) | 6 496 (0.28) | | 2 437 (0.30) |  |
| GAD | 1 893 (0.10) | 695 (0.11) | 1 909 (0.10) | | 698 (0.10) |  |
| any addiction | 1 491 (0.05) | 918 (0.10) | 1 517 (0.06) | | 927 (0.10) |  |
| alcoholism | 569 (0.02) | 368 (0.04) | 575 (0.02) | | 381 (0.04) |  |
| illicit drug addiction | 93 (0.003) | 101 (0.01) | 91 (0.003) | | 104 (0.01) |  |
| OTC/prescription drugs  addiction | 229 (0.01) | 96 (0.01) | 234 (0.01) | | 102 (0.01) |  |
| Ever cannabis | 4 788 (0.17) | 2 780 (0.29) | 4 827 (0.17) | | 2 809 (0.29) |  |
