# Supplementary material for: Genome-wide analysis of self-reported risk-taking behaviour and cross-disorder genetic correlations in the UK Biobank cohort
Source: Transl Psychiatry. 2018 Feb 2;8:39. doi: 10.1038/s41398-017-0079-1 (PMC5804026; doi:10.1038/s41398-017-0079-1)
Supplement: Supplementary file 4 — Supplemental Table 2 [file 41398_2017_79_MOESM4_ESM.docx]

| **Supplementary Table 2: Test-retest numbers and coefficients** | | |  |  |
| --- | --- | --- | --- | --- |
|  | All participants | Mood category | | |
| Risk-taking behaviour |  | Comparison | Mood disorders | Missing |
| Consistent N (frequency) | 16 506 (0.81) | 9 436 (0.82) | 3 113 (0.80) | 3 957 (0.79) |
| Inconsistent N (frequency) | 2 638 (0.13) | 1 432 (0.12) | 579 (0.15) | 627 (0.13) |
| Missing N (frequency) | 1 191 (0.06) | 612 (0.05) | 184 (0.05) | 395 (0.08) |
| total | 20 335 | 11 480 | 3 876 | 4 979 |
| Where: Mood category as per Smith et al, 2013, PLoSOne. Comparison group, no mood disorder; Mood disorder, probable depression or bipolar disorder; Missing, no response or don’t know; | | | | |
