# Supplementary material for: Genome-wide analysis of self-reported risk-taking behaviour and cross-disorder genetic correlations in the UK Biobank cohort
Source: Transl Psychiatry. 2018 Feb 2;8:39. doi: 10.1038/s41398-017-0079-1 (PMC5804026; doi:10.1038/s41398-017-0079-1)
Supplement: Supplementary file 6 — Supplemental Table 4 [file 41398_2017_79_MOESM6_ESM.docx]

| **Supplementary Table 4. Genome-wide significant loci associated with risk-taking in UK Biobank and replication analyses** | | | | | | | | | | | | | |
| --- | --- | --- | --- | --- | --- | --- | --- | --- | --- | --- | --- | --- | --- |
|  |  |  |  | Discovery (n=116 180) | | | | | Replication (n=139474) | | | | |
| SNP | CHR | BP | A1 | BETA | SE | L95 | U95 | P | BETA | SE | L95 | U95 | P |
| rs10511087 | 3 | 85439136 | A | 0.055 | 0.010 | 0.036 | 0.075 | 3.44E-08 | 0.059 | 0.009 | 0.041 | 0.077 | **1.18E-10** |
| rs1368742 | 3 | 85442208 | A | 0.055 | 0.010 | 0.036 | 0.075 | 3.44E-08 | 0.059 | 0.009 | 0.042 | 0.077 | **7.68E-11** |
| rs1991872 | 3 | 85443793 | G | 0.055 | 0.010 | 0.036 | 0.075 | 3.51E-08 | 0.058 | 0.009 | 0.041 | 0.076 | **1.54E-10** |
| rs12637798 | 3 | 85446776 | A | 0.055 | 0.010 | 0.036 | 0.075 | 2.97E-08 | 0.058 | 0.009 | 0.040 | 0.076 | **1.83E-10** |
| rs4308294 | 3 | 85447830 | A | 0.055 | 0.010 | 0.036 | 0.075 | 3.18E-08 |  |  |  |  |  |
| rs12638482 | 3 | 85454815 | C | 0.055 | 0.010 | 0.035 | 0.074 | 4.66E-08 | 0.058 | 0.009 | 0.040 | 0.076 | **1.73E-10** |
| rs11918899 | 3 | 85458577 | A | 0.055 | 0.010 | 0.035 | 0.074 | 4.52E-08 | 0.058 | 0.009 | 0.040 | 0.076 | **1.68E-10** |
| rs4856569 | 3 | 85464125 | A | 0.055 | 0.010 | 0.036 | 0.075 | 3.42E-08 | 0.058 | 0.009 | 0.041 | 0.076 | **1.50E-10** |
| rs80285517 | 3 | 85467372 | C | 0.056 | 0.010 | 0.037 | 0.076 | 1.87E-08 |  |  |  |  |  |
| rs2053108 | 3 | 85467972 | C | 0.055 | 0.010 | 0.035 | 0.074 | 3.61E-08 | 0.059 | 0.009 | 0.041 | 0.076 | **1.42E-10** |
| rs6808400 | 3 | 85468059 | T | 0.055 | 0.010 | 0.035 | 0.074 | 3.61E-08 | 0.059 | 0.009 | 0.041 | 0.076 | **1.42E-10** |
| rs62250686 | 3 | 85468446 | T | 0.055 | 0.010 | 0.036 | 0.075 | 2.81E-08 |  |  |  |  |  |
| rs6780968 | 3 | 85469041 | C | 0.055 | 0.010 | 0.035 | 0.074 | 3.51E-08 | 0.058 | 0.009 | 0.041 | 0.076 | **1.50E-10** |
| rs62250687 | 3 | 85469353 | T | 0.055 | 0.010 | 0.035 | 0.074 | 4.11E-08 | 0.059 | 0.009 | 0.041 | 0.077 | **1.24E-10** |
| rs60311538 | 3 | 85470687 | T | 0.055 | 0.010 | 0.035 | 0.074 | 3.90E-08 | 0.060 | 0.009 | 0.042 | 0.077 | **6.91E-11** |
| rs144059553 | 3 | 85471325 | AAC | 0.055 | 0.010 | 0.035 | 0.074 | 4.43E-08 |  |  |  |  |  |
| rs7636243 | 3 | 85472033 | C | 0.055 | 0.010 | 0.035 | 0.074 | 3.68E-08 | 0.058 | 0.009 | 0.041 | 0.076 | **1.50E-10** |
| rs7650284 | 3 | 85472227 | T | 0.055 | 0.010 | 0.035 | 0.074 | 3.92E-08 | 0.058 | 0.009 | 0.040 | 0.076 | **1.74E-10** |
| rs7638953 | 3 | 85472462 | G | 0.055 | 0.010 | 0.035 | 0.074 | 4.36E-08 | 0.058 | 0.009 | 0.040 | 0.076 | **1.84E-10** |
| rs4856571 | 3 | 85474868 | G | 0.055 | 0.010 | 0.035 | 0.074 | 4.19E-08 | 0.059 | 0.009 | 0.041 | 0.077 | **7.72E-11** |
| rs35894540 | 3 | 85475647 | T | 0.055 | 0.010 | 0.035 | 0.074 | 3.71E-08 | 0.059 | 0.009 | 0.041 | 0.077 | **1.12E-10** |
| rs11716233 | 3 | 85481659 | T | 0.064 | 0.012 | 0.041 | 0.087 | 3.01E-08 | 0.054 | 0.011 | 0.033 | 0.075 | **3.44E-07** |
| rs35827242 | 3 | 85482661 | G | 0.055 | 0.010 | 0.035 | 0.075 | 3.59E-08 | 0.059 | 0.009 | 0.041 | 0.077 | **1.01E-10** |
| rs35489310 | 3 | 85486209 | G | 0.064 | 0.012 | 0.042 | 0.087 | 2.59E-08 |  |  |  |  |  |
| rs12629036 | 3 | 85488299 | T | 0.054 | 0.010 | 0.035 | 0.074 | 4.77E-08 |  |  |  |  |  |
| rs9874491 | 3 | 85489907 | T | 0.064 | 0.012 | 0.042 | 0.087 | 2.62E-08 |  |  |  |  |  |
| rs2082556 | 3 | 85490947 | G | 0.054 | 0.010 | 0.035 | 0.074 | 4.97E-08 | 0.060 | 0.009 | 0.042 | 0.078 | **6.11E-11** |
| rs9820228 | 3 | 85493753 | T | 0.063 | 0.012 | 0.041 | 0.086 | 3.90E-08 | 0.054 | 0.011 | 0.033 | 0.075 | **3.45E-07** |
| rs2033526 | 3 | 85496758 | A | 0.057 | 0.010 | 0.037 | 0.077 | 1.34E-08 | 0.059 | 0.009 | 0.041 | 0.077 | **9.23E-11** |
| rs9828679 | 3 | 85497198 | C | 0.064 | 0.012 | 0.041 | 0.086 | 3.10E-08 | 0.054 | 0.011 | 0.034 | 0.075 | **2.93E-07** |
| rs145394945 | 3 | 85503999 | C | 0.055 | 0.010 | 0.035 | 0.074 | 4.74E-08 |  |  |  |  |  |
| rs2196098 | 3 | 85505246 | G | 0.064 | 0.012 | 0.041 | 0.086 | 3.65E-08 | 0.055 | 0.011 | 0.034 | 0.075 | **2.60E-07** |
| rs13068434 | 3 | 85508814 | G | 0.064 | 0.012 | 0.041 | 0.086 | 3.42E-08 | 0.054 | 0.011 | 0.034 | 0.075 | **2.95E-07** |
| rs78867021 | 3 | 85510809 | TATG | 0.055 | 0.010 | 0.036 | 0.075 | 3.68E-08 |  |  |  |  |  |
| rs6762267 | 3 | 85513115 | C | 0.055 | 0.010 | 0.035 | 0.074 | 4.37E-08 | 0.059 | 0.009 | 0.041 | 0.077 | **7.87E-11** |
| rs62250712 | 3 | 85513716 | C | 0.055 | 0.010 | 0.035 | 0.074 | 4.67E-08 | 0.060 | 0.009 | 0.042 | 0.077 | **7.00E-11** |
| rs62250713 | 3 | 85513793 | A | 0.056 | 0.010 | 0.037 | 0.076 | 2.13E-08 | 0.060 | 0.009 | 0.042 | 0.078 | **7.97E-11** |
| rs112911909 | 3 | 85517112 | A | -0.057 | 0.010 | -0.077 | -0.037 | 1.66E-08 |  |  |  |  |  |
| rs13070166 | 3 | 85517507 | A | 0.065 | 0.012 | 0.042 | 0.088 | 1.71E-08 | 0.053 | 0.011 | 0.033 | 0.074 | **4.09E-07** |
| rs34133544 | 3 | 85517748 | G | 0.056 | 0.010 | 0.036 | 0.076 | 4.02E-08 |  |  |  |  |  |
| rs62250716 | 3 | 85517752 | G | -0.058 | 0.010 | -0.078 | -0.038 | 1.24E-08 |  |  |  |  |  |
| rs2875907 | 3 | 85518580 | A | 0.056 | 0.010 | 0.036 | 0.076 | 3.16E-08 | 0.062 | 0.009 | 0.044 | 0.080 | **2.79E-11** |
| rs960986 | 3 | 85519305 | T | -0.056 | 0.010 | -0.076 | -0.036 | 2.87E-08 | -0.062 | 0.009 | -0.080 | -0.044 | **2.29E-11** |
| rs144888873 | 3 | 85520967 | T | 0.065 | 0.012 | 0.043 | 0.088 | 1.44E-08 |  |  |  |  |  |
| rs62250717 | 3 | 85521990 | G | -0.055 | 0.010 | -0.075 | -0.035 | 4.82E-08 | -0.063 | 0.009 | -0.081 | -0.045 | **1.03E-11** |
| rs9841144 | 3 | 85531199 | T | 0.064 | 0.011 | 0.042 | 0.087 | 2.46E-08 | 0.053 | 0.011 | 0.032 | 0.074 | **4.85E-07** |
| rs1379778 | 3 | 85536091 | AT | -0.058 | 0.010 | -0.078 | -0.037 | 2.77E-08 |  |  |  |  |  |
| rs9849399 | 3 | 85537665 | C | 0.064 | 0.011 | 0.041 | 0.086 | 2.76E-08 | 0.053 | 0.011 | 0.033 | 0.074 | **4.58E-07** |
| rs56088977 | 3 | 85538811 | G | 0.064 | 0.012 | 0.041 | 0.088 | 4.55E-08 |  |  |  |  |  |
| rs34418561 | 3 | 85538846 | C | 0.064 | 0.011 | 0.041 | 0.086 | 2.63E-08 |  |  |  |  |  |
| rs138246680 | 3 | 85539217 | C | 0.056 | 0.010 | 0.036 | 0.076 | 4.86E-08 |  |  |  |  |  |
| rs9844512 | 3 | 85544070 | A | 0.064 | 0.011 | 0.041 | 0.087 | 2.59E-08 | 0.053 | 0.011 | 0.033 | 0.074 | **4.68E-07** |
| rs76395182 | 3 | 85547337 | G | -0.061 | 0.011 | -0.082 | -0.040 | 1.06E-08 |  |  |  |  |  |
| rs4637303 | 3 | 85552787 | C | 0.056 | 0.010 | 0.036 | 0.075 | 4.42E-08 | 0.062 | 0.009 | 0.044 | 0.080 | **2.38E-11** |
| rs6809805 | 3 | 85553096 | A | 0.064 | 0.012 | 0.042 | 0.087 | 2.13E-08 | 0.053 | 0.011 | 0.032 | 0.074 | **4.72E-07** |
| rs13084531 | 3 | 85553994 | G | 0.067 | 0.012 | 0.044 | 0.089 | 8.75E-09 | 0.053 | 0.011 | 0.033 | 0.074 | **4.07E-07** |
| rs12495758 | 3 | 85554262 | G | 0.055 | 0.010 | 0.036 | 0.075 | 4.62E-08 |  |  |  |  |  |
| rs62250759 | 3 | 85569026 | G | -0.055 | 0.010 | -0.075 | -0.035 | 4.90E-08 | -0.064 | 0.009 | -0.082 | -0.046 | **5.28E-12** |
| rs9841829 | 3 | 85569361 | G | 0.064 | 0.012 | 0.041 | 0.086 | 3.34E-08 | 0.054 | 0.011 | 0.033 | 0.075 | **3.09E-07** |
| rs9851444 | 3 | 85576706 | C | 0.065 | 0.012 | 0.042 | 0.087 | 2.06E-08 | 0.054 | 0.011 | 0.033 | 0.074 | **3.59E-07** |
| rs9873400 | 3 | 85590097 | A | 0.063 | 0.012 | 0.040 | 0.085 | 4.61E-08 | 0.055 | 0.011 | 0.034 | 0.076 | **2.02E-07** |
| rs12492753 | 3 | 85601440 | T | 0.056 | 0.010 | 0.036 | 0.076 | 3.73E-08 | 0.063 | 0.009 | 0.044 | 0.081 | **1.41E-11** |
| rs72585634 | 3 | 85602434 | ATC | 0.057 | 0.010 | 0.037 | 0.077 | 2.28E-08 |  |  |  |  |  |
| rs7652808 | 3 | 85603643 | T | 0.055 | 0.010 | 0.035 | 0.075 | 4.90E-08 | 0.062 | 0.009 | 0.043 | 0.080 | **2.93E-11** |
| rs11713902 | 3 | 85604425 | T | 0.063 | 0.012 | 0.041 | 0.086 | 4.00E-08 | 0.054 | 0.011 | 0.033 | 0.074 | **3.75E-07** |
| rs13077660 | 3 | 85616260 | C | 0.063 | 0.012 | 0.040 | 0.086 | 4.73E-08 | 0.055 | 0.011 | 0.034 | 0.076 | **2.29E-07** |
| rs13353478 | 3 | 85621395 | T | 0.064 | 0.012 | 0.041 | 0.086 | 3.68E-08 | 0.055 | 0.011 | 0.034 | 0.076 | **2.23E-07** |
| rs6790699 | 3 | 85624189 | A | 0.055 | 0.010 | 0.035 | 0.074 | 4.91E-08 | 0.062 | 0.009 | 0.044 | 0.080 | **9.52E-12** |
| rs9379971 | 6 | 27259308 | A | -0.063 | 0.011 | -0.084 | -0.042 | 2.31E-09 | -0.010 | 0.009 | -0.028 | 0.009 | 0.2920 |
| rs7746841 | 6 | 27261700 | G | -0.054 | 0.010 | -0.073 | -0.035 | 3.94E-08 | -0.009 | 0.009 | -0.026 | 0.009 | 0.3170 |
| rs35316606 | 6 | 27267230 | CA | -0.054 | 0.010 | -0.073 | -0.035 | 3.32E-08 |  |  |  |  |  |
| rs2393923 | 6 | 27270261 | C | -0.059 | 0.010 | -0.079 | -0.038 | 1.73E-08 | -0.024 | 0.010 | -0.043 | -0.006 | **0.0108** |
| rs7771953 | 6 | 27271343 | A | -0.055 | 0.010 | -0.074 | -0.036 | 1.88E-08 | -0.010 | 0.009 | -0.028 | 0.007 | 0.2440 |
| rs6456772 | 6 | 27273365 | A | -0.054 | 0.010 | -0.073 | -0.035 | 2.69E-08 | -0.009 | 0.009 | -0.026 | 0.009 | 0.3200 |
| rs3800319 | 6 | 27280407 | G | -0.055 | 0.010 | -0.074 | -0.036 | 2.38E-08 | -0.011 | 0.009 | -0.028 | 0.007 | 0.2270 |
| rs2893911 | 6 | 27283303 | A | -0.054 | 0.010 | -0.074 | -0.035 | 3.25E-08 | -0.011 | 0.009 | -0.028 | 0.007 | 0.2390 |
| rs9379973 | 6 | 27288419 | C | -0.055 | 0.010 | -0.074 | -0.036 | 2.11E-08 |  |  |  |  |  |
| rs112356720 | 6 | 27289497 | ACTTTT | -0.055 | 0.010 | -0.074 | -0.036 | 1.63E-08 |  |  |  |  |  |
| rs6923811 | 6 | 27289776 | C | -0.059 | 0.010 | -0.079 | -0.038 | 1.92E-08 | -0.024 | 0.010 | -0.043 | -0.005 | **0.0119** |
| rs2092114 | 6 | 27290082 | C | -0.055 | 0.010 | -0.074 | -0.036 | 2.04E-08 | -0.007 | 0.009 | -0.025 | 0.010 | 0.4200 |
| rs9368501 | 6 | 27295082 | G | -0.055 | 0.010 | -0.074 | -0.036 | 2.16E-08 | -0.008 | 0.009 | -0.026 | 0.009 | 0.3610 |
