# Supplementary material for: Genome-wide analysis of self-reported risk-taking behaviour and cross-disorder genetic correlations in the UK Biobank cohort
Source: Transl Psychiatry. 2018 Feb 2;8:39. doi: 10.1038/s41398-017-0079-1 (PMC5804026; doi:10.1038/s41398-017-0079-1)
Supplement: Supplementary file 7 — Supplemental Table 5 [file 41398_2017_79_MOESM7_ESM.docx]

| **Supplementary Table 5: Effects of risk-taking PRS on risk-taking behaviour in an independent sample set.** | | | | |
| --- | --- | --- | --- | --- |
| P threshold | OR | 95% CI | P | R2 |
| 1x10^-5^ | 1.015 | 1.011 - 1.020 | <0.001 | 0.034 |
| 0.001 | 1.040 | 1.035 - 1.044 | <0.001 | 0.035 |
| 0.05 | 1.055 | 1.050 - 1.060 | <0.001 | 0.037 |
| Where models included age, sex, chip, PCAs1-8 and the PRS. | | | | |
