# Supplementary material for: Genome-wide analysis of self-reported risk-taking behaviour and cross-disorder genetic correlations in the UK Biobank cohort
Source: Transl Psychiatry. 2018 Feb 2;8:39. doi: 10.1038/s41398-017-0079-1 (PMC5804026; doi:10.1038/s41398-017-0079-1)
Supplement: Supplementary file 8 — Supplemental Table 6 [file 41398_2017_79_MOESM8_ESM.docx]

| **Supplementary Table 6: Variant Effect Predictor summary of impact of *CADM2* SNPs on the CADM2 protein** | | | | | |
| --- | --- | --- | --- | --- | --- |
| SNP | Allele | Consequence | Impact | Gene type | Feature |
| rs10511087 | G | intron_variant | MODIFIER | protein_coding | ENST00000383699 |
| rs1368742 | G | intron_variant | MODIFIER | protein_coding | ENST00000383699 |
| rs1991872 | A | intron_variant | MODIFIER | protein_coding | ENST00000383699 |
| rs12637798 | G | intron_variant | MODIFIER | protein_coding | ENST00000383699 |
| rs4308294 | T | intron_variant | MODIFIER | protein_coding | ENST00000383699 |
| rs12638482 | T | intron_variant | MODIFIER | protein_coding | ENST00000383699 |
| rs4856569 | G | intron_variant | MODIFIER | protein_coding | ENST00000383699 |
| rs80285517 | ATT | intron_variant | MODIFIER | protein_coding | ENST00000383699 |
| rs2053108 | A | intron_variant | MODIFIER | protein_coding | ENST00000383699 |
| rs6808400 | C | intron_variant | MODIFIER | protein_coding | ENST00000383699 |
| rs62250686 | A | intron_variant | MODIFIER | protein_coding | ENST00000383699 |
| rs6780968 | G | intron_variant | MODIFIER | protein_coding | ENST00000383699 |
| rs62250687 | A | intron_variant | MODIFIER | protein_coding | ENST00000383699 |
| rs60311538 | G | intron_variant | MODIFIER | protein_coding | ENST00000383699 |
| rs144059553 | - | intron_variant | MODIFIER | protein_coding | ENST00000383699 |
| rs7636243 | T | intron_variant | MODIFIER | protein_coding | ENST00000383699 |
| rs7650284 | C | intron_variant | MODIFIER | protein_coding | ENST00000383699 |
| rs7638953 | A | intron_variant | MODIFIER | protein_coding | ENST00000383699 |
| rs4856571 | C | intron_variant | MODIFIER | protein_coding | ENST00000383699 |
| rs35894540 | C | intron_variant | MODIFIER | protein_coding | ENST00000383699 |
| rs11716233 | T | intron_variant | MODIFIER | protein_coding | ENST00000383699 |
| rs35827242 | A | intron_variant | MODIFIER | protein_coding | ENST00000383699 |
| rs35489310 | - | intron_variant | MODIFIER | protein_coding | ENST00000383699 |
| rs12629036 | C | intron_variant | MODIFIER | protein_coding | ENST00000383699 |
| rs9874491 | T | intron_variant | MODIFIER | protein_coding | ENST00000383699 |
| rs2082556 | A | intron_variant | MODIFIER | protein_coding | ENST00000383699 |
| rs9820228 | T | intron_variant | MODIFIER | protein_coding | ENST00000383699 |
| rs2033526 | C | intron_variant | MODIFIER | protein_coding | ENST00000383699 |
| rs9828679 | C | intron_variant | MODIFIER | protein_coding | ENST00000383699 |
| rs145394945 | AT | intron_variant | MODIFIER | protein_coding | ENST00000383699 |
| rs2196098 | G | intron_variant | MODIFIER | protein_coding | ENST00000383699 |
| rs13068434 | G | intron_variant | MODIFIER | protein_coding | ENST00000383699 |
| rs78867021 | - | intron_variant | MODIFIER | protein_coding | ENST00000383699 |
| rs6762267 | A | intron_variant | MODIFIER | protein_coding | ENST00000383699 |
| rs62250712 | T | intron_variant | MODIFIER | protein_coding | ENST00000383699 |
| rs62250713 | G | intron_variant | MODIFIER | protein_coding | ENST00000383699 |
| rs112911909 | A | intron_variant | MODIFIER | protein_coding | ENST00000383699 |
| rs13070166 | A | intron_variant | MODIFIER | protein_coding | ENST00000383699 |
| rs34133544 | A | intron_variant | MODIFIER | protein_coding | ENST00000383699 |
| rs62250716 | G | intron_variant | MODIFIER | protein_coding | ENST00000383699 |
| rs2875907 | G | intron_variant | MODIFIER | protein_coding | ENST00000383699 |
| rs960986 | T | intron_variant | MODIFIER | protein_coding | ENST00000383699 |
| rs144888873 | - | intron_variant | MODIFIER | protein_coding | ENST00000383699 |
| rs62250717 | G | intron_variant | MODIFIER | protein_coding | ENST00000383699 |
| rs9841144 | T | intron_variant | MODIFIER | protein_coding | ENST00000383699 |
| rs9849399 | C | intron_variant | MODIFIER | protein_coding | ENST00000383699 |
| rs56088977 | - | intron_variant | MODIFIER | protein_coding | ENST00000383699 |
| rs34418561 | - | intron_variant | MODIFIER | protein_coding | ENST00000383699 |
| rs138246680 | ATTAT | intron_variant | MODIFIER | protein_coding | ENST00000383699 |
| rs9844512 | A | intron_variant | MODIFIER | protein_coding | ENST00000383699 |
| rs76395182 | G | intron_variant | MODIFIER | protein_coding | ENST00000383699 |
| rs4637303 | T | intron_variant | MODIFIER | protein_coding | ENST00000383699 |
| rs6809805 | A | intron_variant | MODIFIER | protein_coding | ENST00000383699 |
| rs13084531 | G | intron_variant | MODIFIER | protein_coding | ENST00000383699 |
| rs12495758 | A | 5_prime_UTR_variant | MODIFIER | protein_coding | NM_001256502.1 |
| rs62250759 | G | intron_variant | MODIFIER | protein_coding | ENST00000383699 |
| rs9841829 | G | intron_variant | MODIFIER | protein_coding | ENST00000383699 |
| rs9851444 | C | intron_variant | MODIFIER | protein_coding | ENST00000383699 |
| rs9873400 | A | intron_variant | MODIFIER | protein_coding | ENST00000383699 |
| rs72585634 | - | intron_variant | MODIFIER | protein_coding | ENST00000383699 |
| rs7652808 | G | intron_variant | MODIFIER | protein_coding | ENST00000383699 |
| rs11713902 | T | intron_variant | MODIFIER | protein_coding | ENST00000383699 |
| rs13077660 | C | intron_variant | MODIFIER | protein_coding | ENST00000383699 |
| rs13353478 | T | intron_variant | MODIFIER | protein_coding | ENST00000383699 |
| rs6790699 | G | intron_variant | MODIFIER | protein_coding | ENST00000383699 |
