# Supplementary material for: Genome-wide analysis of self-reported risk-taking behaviour and cross-disorder genetic correlations in the UK Biobank cohort
Source: Transl Psychiatry. 2018 Feb 2;8:39. doi: 10.1038/s41398-017-0079-1 (PMC5804026; doi:10.1038/s41398-017-0079-1)
Supplement: Supplementary file 9 — Supplemental Table 7 [file 41398_2017_79_MOESM9_ESM.docx]

| **Supplementary Table 7: Genes in the *CADM2* locus** | | | | | | |
| --- | --- | --- | --- | --- | --- | --- |
| **Gene** | **EntrezID** | **Name (GeneCards)** | **Localisation (GeneCards)** | **Gene ontology (GeneCards)** | **Gene sumamry (Entrez)** | **Gene expression pattern (GTEx )** |
| *CADM2*^* | 253559 | cell adhesion molecule 2 | plasma membrane | Cell adhesion binding, receptor activity, receptor binding, protein homodimerisation activity | synaptic cell adhesion molecule/immunoglobulin superfamily | low expression, except in brain |
| *CADM2-AS2* | 100874037 | CADM2 Antisense RNA 2 | NA | NA | NA | NA |
| *miR5688* | 100847077 | NA | NA | NA | NA | NA |
| Where: NA, not available: * genes highlighted by eQTL analysis in brain: ^ genes highlighted by VEP | | | | | | |
