# Supplementary material for: Genome-wide analysis of self-reported risk-taking behaviour and cross-disorder genetic correlations in the UK Biobank cohort
Source: Transl Psychiatry. 2018 Feb 2;8:39. doi: 10.1038/s41398-017-0079-1 (PMC5804026; doi:10.1038/s41398-017-0079-1)
Supplement: Supplementary file 10 — Supplemental Table 8 [file 41398_2017_79_MOESM10_ESM.docx]

| **Supplementary Table 8: Previously reported signals in the *CADM2* locus and results in the risk-taking GWAS** | | | | | | | | | | |  |
| --- | --- | --- | --- | --- | --- | --- | --- | --- | --- | --- | --- |
| **GWAS catalogue** | | | | **UK BioBank Risk-taking behaviour GWAS (n=116 255)** | | | | | | | |
| **locus** | **trait** | **SNP-allele** | **PMID** | **SNP** | **A1** | **MAF** | **BETA** | **SE** | **L95** | **U95** | **P** |
| 3p12.1 | ADHD | rs7642644-T | 26174813 | rs7642644 | C | 0.14 | -0.025 | 0.014 | -0.053 | 0.003 | 8.57E-02 |
| 3p12.1 | visceral adiposity | rs13323436-A | 22589738 | rs13323436 | A | 0.07 | 0.069 | 0.018 | 0.033 | 0.105 | 1.92E-04 |
| 3p12.1 | longevity | rs9841144-? | 25199915 | rs9841144 | T | 0.23 | **0.064** | **0.011** | **0.042** | **0.087** | **2.46E-08** |
| 3p12.1 | cognitive function, information processing | rs17518584-T | 25644384, 25869804 | rs17518584 | C | 0.38 | 0.053 | 0.010 | 0.034 | 0.073 | 1.17E-07 |
| 3p12.1 | educational attainment | rs55686445-C | 27046643 | rs55686445 | C | 0.35 | -0.046 | 0.010 | -0.067 | -0.026 | 6.76E-06 |
| 3p12.1 | BMI | rs13078960-G | 25673413 | rs13078960 | G | 0.20 | 0.027 | 0.012 | 0.003 | 0.051 | 2.73E-02 |
| 3p12.1 | temperament | rs12494658-T | 22832960 | rs12494658 | C | 0.24 | -0.012 | 0.011 | -0.034 | 0.011 | 3.12E-01 |
| 3p12.1 | BMI, obesity | rs13078807-G | 20935630, 23563607 | rs13078807 | G | 0.20 | 0.027 | 0.012 | 0.003 | 0.051 | 2.49E-02 |
| 3p12.1 | alzhiemers | rs71316816-C | 25778476 | rs71316816 | T | 0.07 | 0.020 | 0.019 | -0.017 | 0.058 | 2.89E-01 |
| 3p12.1 | educational attainment | rs112374913-? | 27046643 | rs112374913 | A | 0.40 | -0.014 | 0.010 | -0.033 | 0.006 | 1.70E-01 |
| 3p12.1 | subq adipose | rs2324999-T | 22589738 | rs2324999 | T | 0.20 | 0.019 | 0.012 | -0.005 | 0.043 | 1.27E-01 |
| 3p12.1 | educational attainment | rs56262138-A | 27225129 | rs56262138 | A | 0.30 | -0.025 | 0.011 | -0.047 | -0.004 | 2.10E-02 |
| 3p12.1 | educational attainment | rs62263923-A | 27225129 | na |  |  |  |  |  |  |  |
| 3p12.1 | age first sexual intercourse | rs12714592-A | 27089180 | rs12714592 | C | 0.27 | 0.015 | 0.011 | 0.036 | 1.365 | 1.72E-01 |
| 3p12.1 | age first sexual intercourse | rs57401290- GGTGTGT | 27089180 | na |  |  |  |  |  |  |  |
| 3p12.1 | risk propensity | rs4856591 | 27089180 | na |  |  |  |  |  |  |  |
| 3p12.1 | alcohol consumption | rs9841829-G | * | rs9841829-G | G | 0.23 | **0.064** | **0.012** | **0.041** | **0.086** | **3.34E-08** |
| *https://doi.org/10.1101/116707 | | |  |  |  |  |  |  |  |  |  |
