# Supplementary material for: Genome-wide analysis of self-reported risk-taking behaviour and cross-disorder genetic correlations in the UK Biobank cohort
Source: Transl Psychiatry. 2018 Feb 2;8:39. doi: 10.1038/s41398-017-0079-1 (PMC5804026; doi:10.1038/s41398-017-0079-1)
Supplement: Supplementary file 11 — Supplemental Table 9 [file 41398_2017_79_MOESM11_ESM.docx]

| **Supplemental Table 9: Association of risk-taking loci with psychiatric traits** | | | | | | | | | |  |  |  |  |  |  |  |  |  |  |  |
| --- | --- | --- | --- | --- | --- | --- | --- | --- | --- | --- | --- | --- | --- | --- | --- | --- | --- | --- | --- | --- |
| locus: signal |  |  | risk-taking | | | ADHD | | | SCZ | | | PTSD | | | BPD | | | MDD | | |
|  | SNP | A1 | OR | SE | P | OR | SE | P | OR | SE | P | OR | SE | P | OR | SE | P | OR | SE | P |
| chr3:1 | rs13084531* | C | 0.97 | 0.01 | **8.75E-09** | 0.97 | 0.02 | 0.0998 | 1.02 | 0.01 | 0.1246 | 1.00 | 0.04 | 0.9622 |  |  |  |  |  |  |
|  | rs13077660 | A | 0.97 | 0.01 | **4.73E-08** |  |  |  |  |  |  |  |  |  | 0.03 | 0.47 | 0.9978 | 0.98 | 0.03 | 0.5275 |
| chr3:2 | rs62250716* | A | 1.03 | 0.01 | **1.24E-08** | 1.03 | 0.01 | **0.0115** |  |  |  |  |  |  |  |  |  |  |  |  |
|  | rs112911909 | G | 1.02 | 0.01 | **1.66E-08** |  |  |  | 0.99 | 0.01 | 0.2834 | 0.99 | 0.04 | 0.8612 |  |  |  |  |  |  |
|  | rs960986 | C | 1.02 | 0.01 | **2.87E-08** |  |  |  |  |  |  |  |  |  | 0.02 | 0.07 | 0.9823 |  |  |  |
| Where: * indicates lead SNP in the locus; proxies for the lead SNP were chosen from the conditional analysis. | | | | | | | | | | | | | | |  |  |  |  |  |  |
