# Supplementary material for: Genome-wide analysis of self-reported risk-taking behaviour and cross-disorder genetic correlations in the UK Biobank cohort
Source: Transl Psychiatry. 2018 Feb 2;8:39. doi: 10.1038/s41398-017-0079-1 (PMC5804026; doi:10.1038/s41398-017-0079-1)

Supplementary Figure 1: Linkage disequilibrium (LD) structure of the chr 3 locus. Colours and numbers reflect R^2 measures of LD.

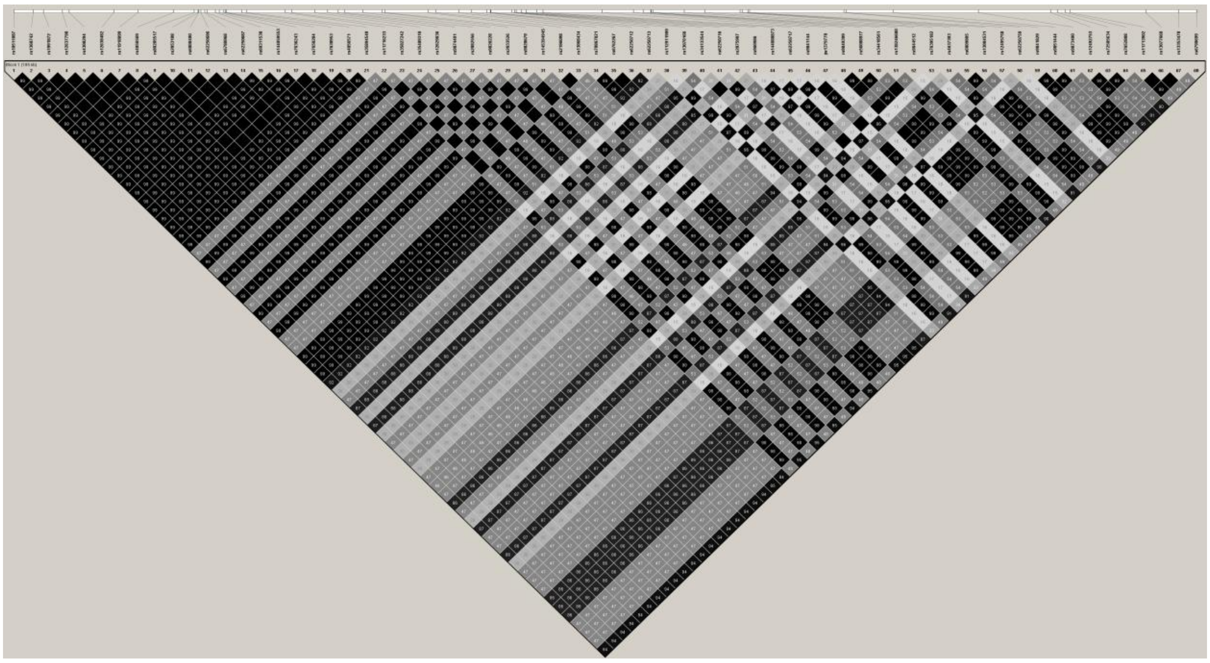

Supplement: Supplementary file 12 — Supplemental Figure 1 [file 41398_2017_79_MOESM12_ESM.pdf]
