# Supplementary figures and images for: Genome-wide analysis of self-reported risk-taking behaviour and cross-disorder genetic correlations in the UK Biobank cohort
Source: Transl Psychiatry. 2018 Feb 2;8:39. doi: 10.1038/s41398-017-0079-1 (PMC5804026; doi:10.1038/s41398-017-0079-1)

Supplementary Figure 3

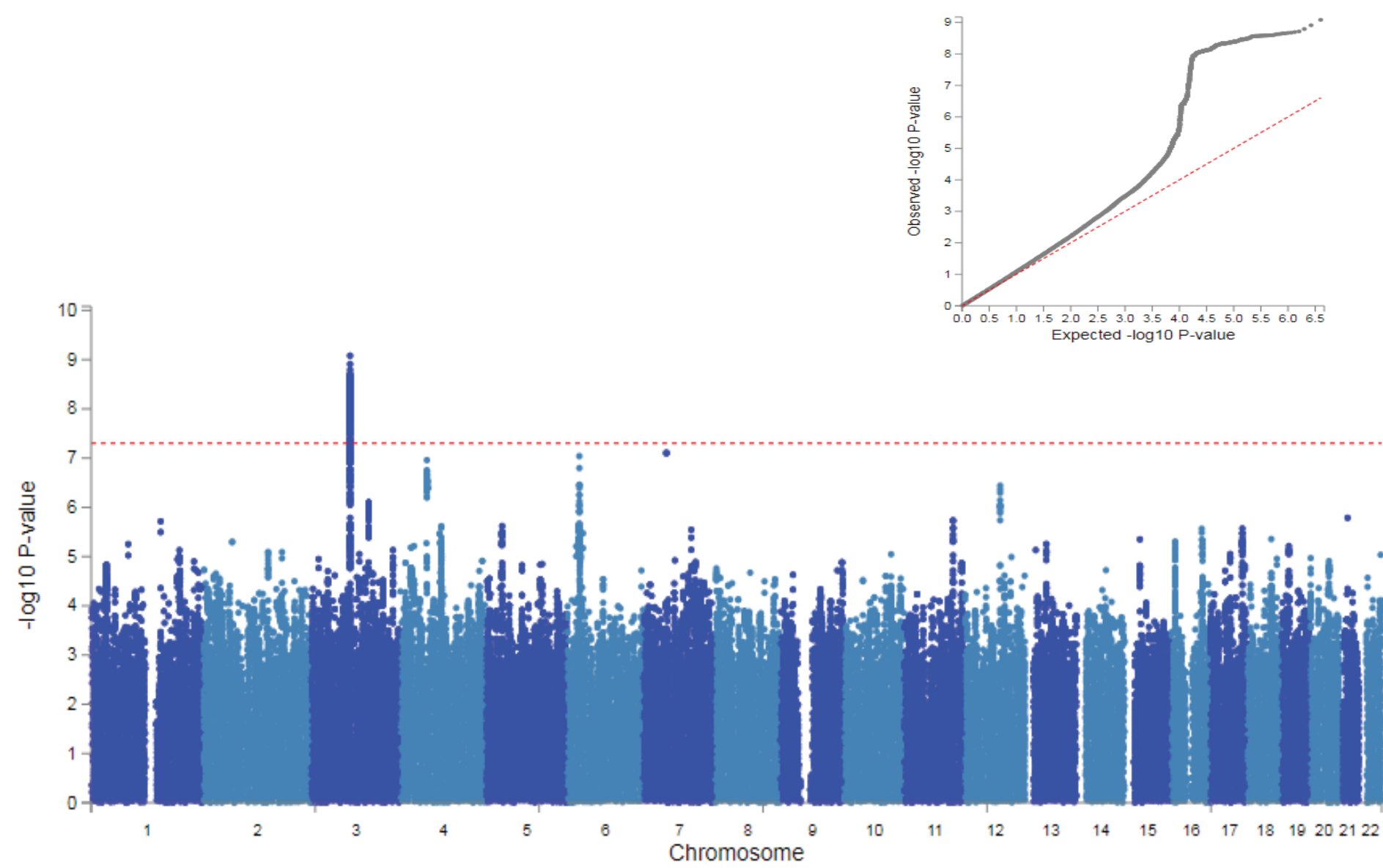

Supplement: Supplementary file 14 — Supplemental Figure 3 [file 41398_2017_79_MOESM14_ESM.pdf]

Supplementary Figure 4

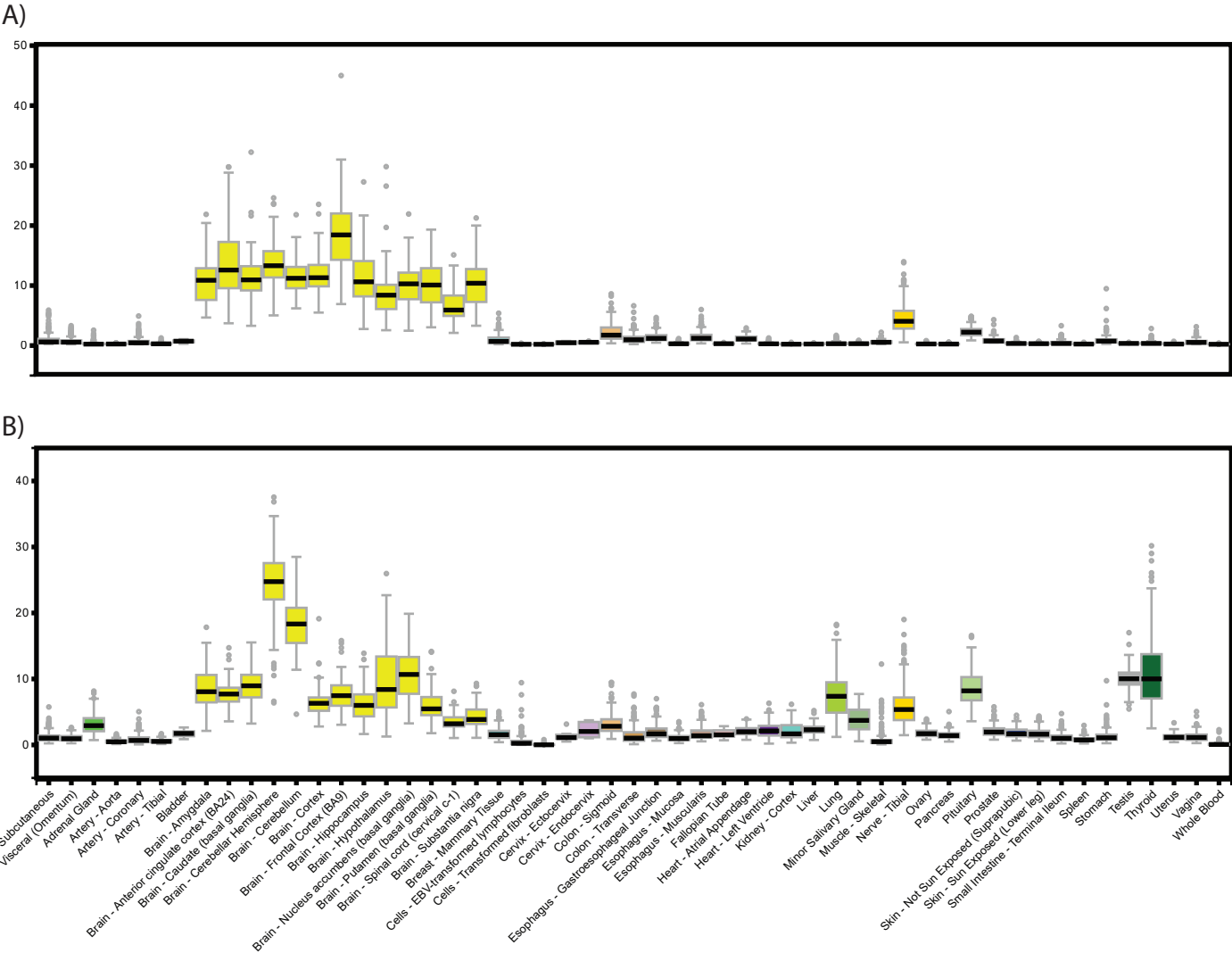

Supplement: Supplementary file 15 — Supplemental Figure 4 [file 41398_2017_79_MOESM15_ESM.pdf]

Supplmentary Figure 5

A)

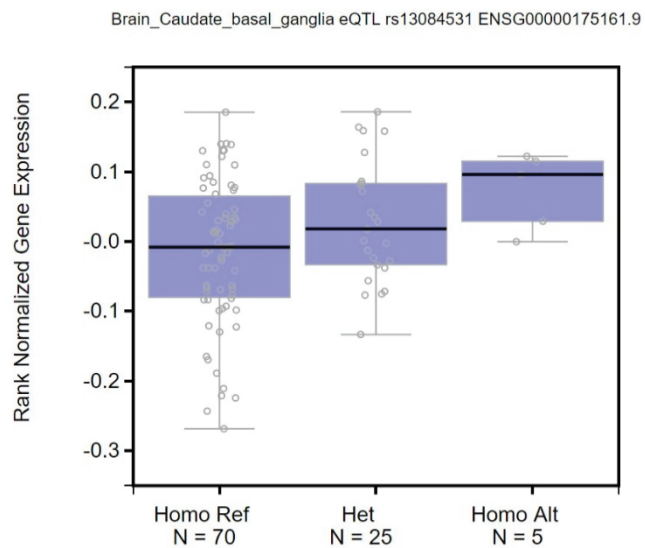

B)

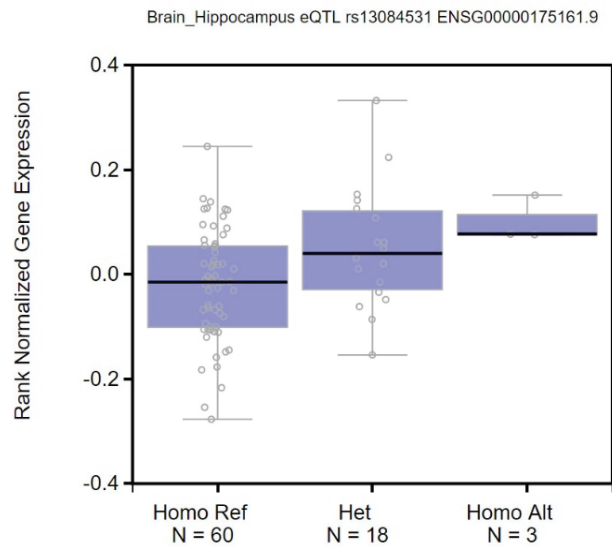

C)

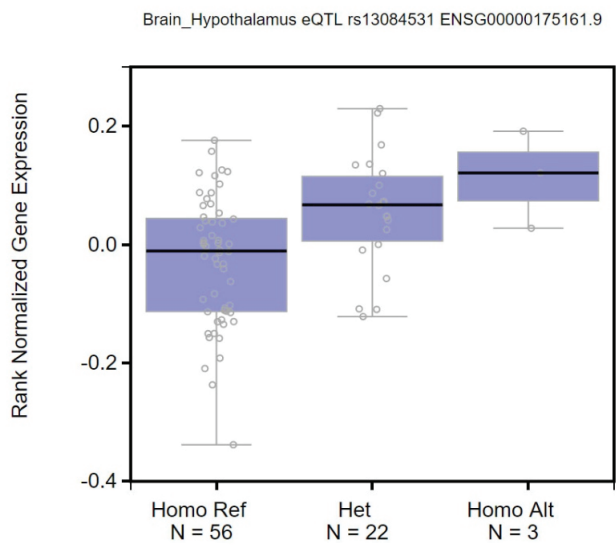

D)

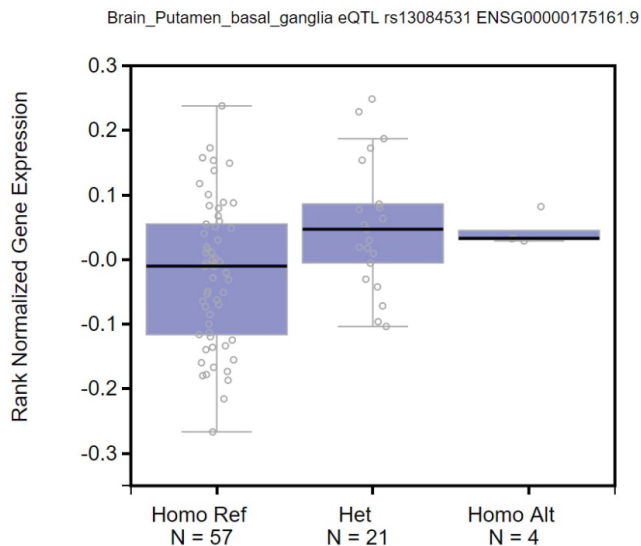

Supplement: Supplementary file 16 — Supplemental Figure 5 [file 41398_2017_79_MOESM16_ESM.pdf]
